# High‐Throughput Data Generation and Transfer Learning Enabled Microstructure‐Property Integrated Design of Nickel‐Based Powder Metallurgy Superalloy

**DOI:** 10.1002/advs.202524365

**Published:** 2026-04-15

**Authors:** Zixin Li, Hongtao Zhang, Zichao Peng, Yuheng Zhang, Qian Lv, Yihuan Cao, Xuqing Wang, Huadong Fu, Jianxin Xie

**Affiliations:** ^1^ Beijing Advanced Innovation Center for Materials Genome Engineering School of Advanced Materials Innovation University of Science and Technology Beijing Beijing China; ^2^ Beijing Key Laboratory of Materials Intelligent Technology School of Advanced Materials Innovation University of Science and Technology Beijing Beijing China; ^3^ Beijing Laboratory of Metallic Materials and Processing for Modern Transportation School of Advanced Materials Innovation University of Science and Technology Beijing Beijing China; ^4^ Institute of Materials Intelligent Technology Liaoning Academy of Materials Shenyang China; ^5^ Science and Technology on Advanced High Temperature Structural Materials Laboratory Beijing Institute of Aeronautical Materials AECC Beijing China

**Keywords:** alloy design, CALPHAD, diffusion‐multiple, nickel‐based powder metallurgy superalloys, transfer learning

## Abstract

Nickel‐based powder metallurgy (PM) superalloys are indispensable for aero‐engine turbine disks, but their design is constrained by complex multicomponent interactions and the difficulty in predicting long‐term service performance. Herein, we present a data‐driven framework integrating high‐throughput thermodynamic calculations, diffusion‐multiple experiments, and transfer learning to predict long‐term microstructural stability and mechanical properties. By calibrating computational models with sparse experimental data, our transfer learning approach enables accurate prediction of microstructural features, which further serve as inputs for mechanical property modeling. From a screening of 10^5^ compositions, we identify a promising low‐density (8.33 g/cm^3^) alloy, designated USTB‐PM750. This alloy exhibits a yield strength of 1138 MPa and a creep life of 141 h to 0.2% strain at 750°C under 480 MPa. Microstructural analysis reveals that its superior performance stems from a low stacking‐fault energy, which promotes the formation of stacking faults and microtwins. These defects subsequently evolve into dense networks of Lomer‐Cottrell locks, further reinforced by solute‐segregation‐induced local phase transformations. This approach significantly improves alloy design efficiency and offers a promising high‐performance candidate material for turbine‐disk applications in advanced aero‐engines.

## Graphical abstract

An integrated transfer learning framework integrates CALPHAD simulations, diffusion‐multiple experiments, and literature data to predict long‐term microstructural stability and short‐term mechanical properties of Ni‐based powder metallurgy superalloys. Based on these model predictions, a high‐performance, low‐density alloy, USTB‐PM750, is designed from a candidate composition space exceeding 2 × 10^5^ alloys.

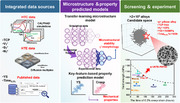

## 1 Introduction

Nickel‐based powder metallurgy (PM) superalloys exhibit exceptional high‐temperature performance and are the prime materials for high‐pressure turbine disks in advanced aero‐engines [1, 2, 3]. These disks must operate for long periods under extreme service conditions, demanding high levels of creep and fatigue resistance, along with other long‐term service properties. Consequently, accurate assessment of such properties is critical for the design of high‐performance alloys. However, the long duration and high cost of these tests result in a severe scarcity of reliable data for alloy development. In addition, PM superalloys typically contain over 10 alloying elements, and the vast compositional space, compounded by complex multicomponent interactions [4, 5], further complicates the alloy design process.

CALculation of PHAse Diagrams (CALPHAD) methods are widely used to facilitate the design of superalloys, as they allow the prediction of phase transformation temperatures [6], precipitate behavior [7], and even mechanical [8] or oxidation‐related properties [9]. Using this approach, the RR1000 PM superalloy was designed by systematically evaluating γ′ volume fraction and solvus temperature, γ/γ′ partitioning, and topologically close‐packed (TCP) phase formation to optimize Al, Ti, Ta, Nb, and Co contents [10]. Nevertheless, CALPHAD accuracy is fundamentally constrained by thermodynamic databases: predictions are reliable only for compositions near established systems but become unreliable for novel alloys [6, 11, 12]. Furthermore, it lacks the capability to predict long‐term service performance.

In recent years, advances in artificial intelligence have enabled machine learning to play an increasingly central role in materials research, offering powerful tools to deliver accurate predictions of microstructural features and mechanical properties [13, 14, 15]. For example, a previous study trained artificial neural network models using alloy compositions as inputs to predict yield strength, tensile strength, elongation, and other key properties, thereby guiding the design of new PM superalloys with performance surpassing that of commercial alloys such as RR1000 [16]. However, the accuracy of machine‐learning models is fundamentally constrained by the scale and quality of available data [17, 18]. Owing to the time and cost of testing, the dataset of long‐term service properties is usually sparse and scattered, making it challenging to build reliable models directly targeting service performance. In contrast, datasets for microstructural characterization [19, 20], tensile strength, and short‐term creep life are relatively abundant [21, 22, 23, 24]. These data can support the modeling of alloy microstructure and short‐term properties.

Long‐term microstructural stability and strong short‐term mechanical performance are both critical for PM nickel‐based superalloy reliability. Microstructural instability is commonly associated with deleterious TCP phase precipitation and destabilization of the γ′ phase during long‐term service, both of which can degrade high‐temperature performance. In particular, TCP phases act as brittle crack initiators that induce creep and fatigue failure [25]. Conversely, where deformation mechanisms remain stable, short‐term high‐temperature properties can predict long‐term behavior, as exemplified by the Larson‐Miller parameter for creep life estimation [26, 27]. Thus, integrating microstructural stability and short‐term mechanical properties as joint design objectives in a machine‐learning framework could accelerate the development of high‐performance PM superalloys.

This study proposes a transfer learning framework for integrated design of microstructure and mechanical properties in PM nickel‐based superalloys. The framework calibrates high‐throughput thermodynamic simulations with limited experimental data to accurately predict the long‐term microstructure. Microstructural stability is then assessed based on whether TCP phases precipitate at the service temperature. These microstructural features are then used as input features in an interpretable model for short‐term mechanical properties, while multi‐objective hierarchical screening with a voting‐based strategy enables coordinated optimization. Using this approach, a low‐density superalloy was successfully designed with stable microstructure, high strength, and excellent creep resistance at 750°C, demonstrating superior performance compared to previously reported PM superalloys.

## 2 Design Strategy

**FIGURE 1:**
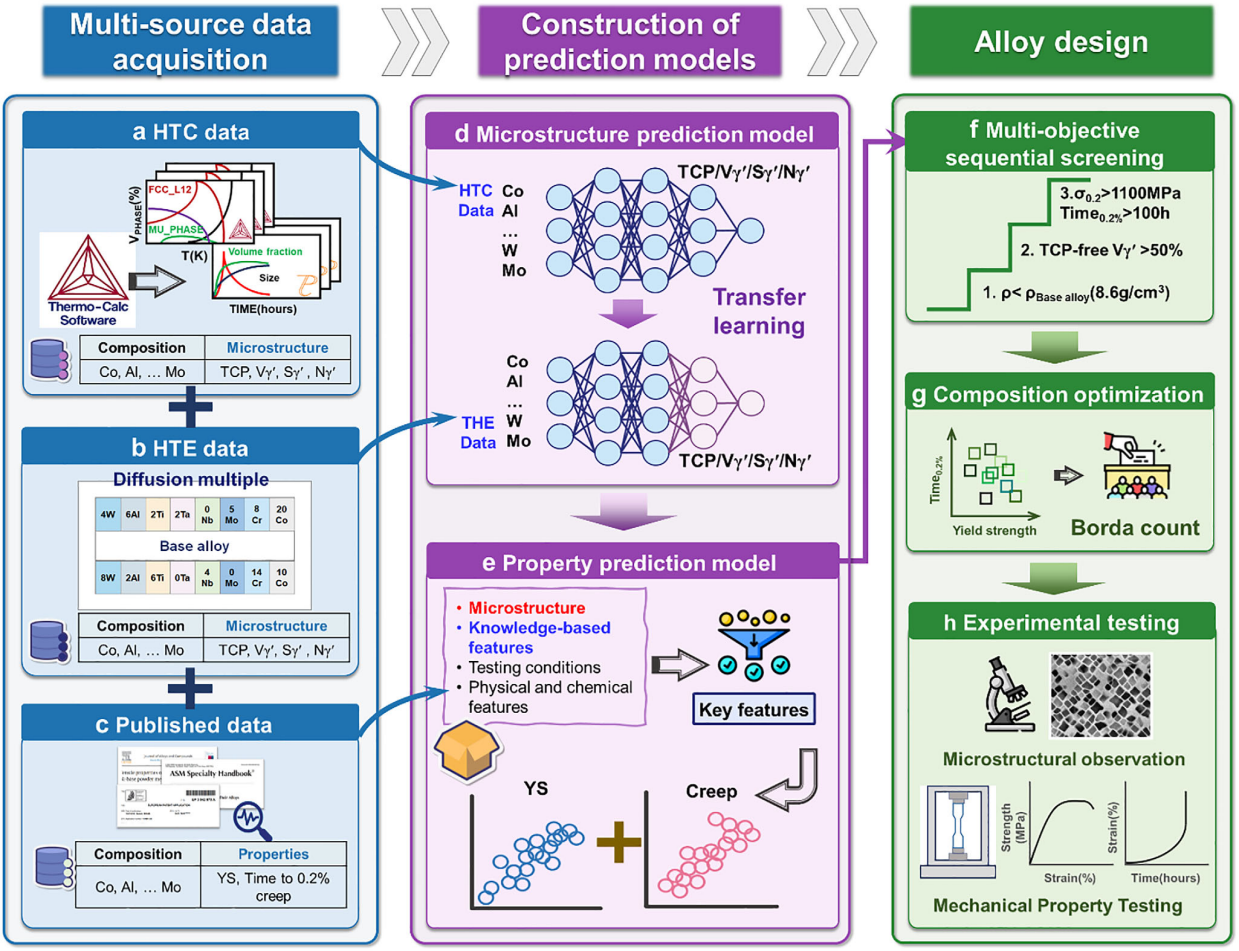
Schematic of the integrated strategy for accelerated superalloy design, combining HTC, HTE, and TL. The framework comprises three main stages: multi‐source data acquisition, model development, and alloy design. (a) Data generation using the CALPHAD method. (b) Experimental data acquisition via diffusion‐multiple. (c) Collection of published data from the literature, patents, and handbooks. (d) Establishing long‐term microstructure prediction models via TL. (e) Establishing short‐term property prediction models. (f) Hierarchical screening of high‐performance alloy compositions. (g) Selection of the optimal composition using the Borda count voting rule. (h) Experimental validation.

The alloy design strategy is illustrated in Figure 1 and comprises three stages: multi‐source data construction, model development, and alloy screening. The dataset integrates high‐throughput calculations (HTC), experiments (HTE), and published literature. Compositional and microstructural data were obtained from CALPHAD‐based HTC and diffusion‐multiple experiments, while short‐term mechanical properties were compiled from literature on Ni‐based PM superalloys. The HTC dataset captures compositional trends governing phase stability and the γ′ features despite computational deviations, whereas the HTE dataset, though smaller, provides more accurate microstructural information. To leverage these complementary characteristics, we adopt a transfer learning approach: first, pre‐train a deep neural network on the HTC dataset, then freeze all layers before the last hidden layer, append and retrain new hidden and output layers on the HTE data. This strategy corrects computational errors and enables reliable microstructural predictions. For short‐term mechanical properties, composition alone proves insufficient to capture performance‐governing features. Therefore, microstructural predictions from the transfer learning (TL) model are combined with knowledge‐based features, testing conditions, and elemental attributes. Following feature selection, machine‐learning models are trained to predict yield strength and the time of 0.2% creep strain (Time_0.2%_), as shown in Figure 1e. A hierarchical, multi‐objective screening process was employed to rank candidate alloys using a Borda count scheme, thereby identifying the most promising Ni‐based PM superalloy composition for turbine‐disk service at 750°C.

### 2.1 Data Acquisition

An HTC dataset was generated by constructing an alloy design space from the third‐generation Ni‐based PM superalloy Alloy 10 composition range (Table S1) and calculating phase constitution and γ′ volume fraction (*V*_γ′_), γ′ size (*S*_γ′_), and number density (*N*_γ′_) at 750°C for all compositions using Thermo‐Calc software. To reduce experimental variability, diffusion‐multiples were employed to acquire the experimental data. Diffusion‐multiples were designed and fabricated on the base alloy Alloy 10 (Ni‐15Co‐11Cr‐2.5Mo‐6W‐3.8Al‐3.9Ti‐0.8Ta‐1.7Nb), as illustrated in Figure S1. Individual alloys for each diffusion couple were synthesized via arc melting, subsequently sectioned, and machined to the specified dimensions. To prevent oxidation during thermal exposures, the diffusion‐multiples were encapsulated in a Ni shell via vacuum electron‐beam welding. The encapsulated samples underwent hot isostatic pressing at 1140°C/150 MPa for 5 h, followed by solution treatment at 1200°C for 150 h (air cooled), and long‐term aging at 750°C for 1000 h (water cooled), thereby generating continuous composition gradients with concomitant microstructural variations. The volume fraction and morphology of the precipitated phases in these samples were statistically analyzed, thereby establishing the HTE dataset. Published data from the literature on Ni‐based PM superalloys (compositions, yield strengths, and times of 0.2% creep strain) were collected using a relatively strict screening and preprocessing workflow. Further details are provided in the Supporting Information.

### 2.2 Model

#### 2.2.1 Microstructure Prediction Model

A transfer learning approach was employed to develop predictive models for long‐term microstructural stability at 750°C, comprising a binary classification model for TCP phase precipitation and three regression models for γ′ characteristics (*V*_γ′_, *S*_γ′_, and *N*_γ′_). Specifically, all alloying elements except Ni were used as inputs. The prediction target was defined as either whether TCP precipitation occurs, encoded as 0 when the volume fraction of TCP phases (V_TCP_) exceeded 1% and 1 otherwise, or one of the γ′ features, including *V*_γ′_, *S*_γ′_, and *N*_γ′_, after aging at 750°C/1000 h. The HTC and HTE datasets were split into training and test sets at a ratio of 8:2. Inputs were standardized (StandardScaler) and regression outputs normalized (Min‐Max). The classification models were constructed with ReLU‐activated hidden layers, a sigmoid output layer, binary cross‐entropy loss, and accuracy and recall as evaluation metrics. Regression models used ReLU throughout with mean absolute percentage error (MAPE) as loss and metric. All models were optimized using the Adam optimizer, coupled with Early Stopping and Reduce Learning Rate on Plateau strategies. The maximum number of epochs was initially set to 3000, and training was terminated if the validation loss did not improve for 10 consecutive epochs, thereby reducing the risk of overfitting. The source domain network comprised input, hidden, and output layers, and was pretrained on the HTC training set, where hyperparameters such as learning rate, number of hidden layers, number of neurons, and batch size were optimized via Optuna. During transfer to HTE data, all pretrained hidden layers except the last one were frozen, and new hidden layers together with the corresponding output layer were appended; the unfrozen last hidden layer and the newly added layers were then retrained on the target domain data, with the remaining trainable hyperparameters further optimized using Optuna. Training parameters are detailed in Table S2.

(1) $$Accuracy=\frac{TP+TN}{TP+FP+TN+FN}$$

(2) $$Recall=\frac{TP}{TP+FN}$$

(3) $$MAPE=\frac{100\%}{n}\sum_{i=1}^{n}\left|\frac{{\hat{y}}_{i}-y_{i}}{y_{i}}\right|$$

where TP, TN, FP, and FN denote true positives, true negatives, false positives, and false negatives, respectively. *y_i_* is the experimental value, ${\hat{y}}_{i}$ is the model prediction value, n is the number of data points.

#### 2.2.2 Property Prediction Model

To establish a model with both strong predictive capability and good interpretability, information related to deformation mechanisms was incorporated into the model. Specifically, microstructural predictions from the TL model were combined with knowledge‐based features, testing conditions, and elemental attributes to construct material descriptors via compositional means and variances. The feature set is detailed in the Table S3. A three‐step screening approach [28] (Pearson correlation filtering (eliminating features with |r| > 0.9), recursive feature elimination, exhaustive optimization) selected the key features. Support vector regression (SVR) was used to model yield strength and ln(time of 0.2% creep strain). The dataset was first split into training and test sets at a ratio of 8:2, and the model hyperparameters were optimized by ten‐fold cross‐validation on the training set. Model accuracy was evaluated using MAPE. For the prediction of new alloy compositions, a bootstrap resampling strategy was adopted, where the training dataset was resampled with replacement 100 times, and the final prediction was obtained as the mean of the resulting predictions, with the standard deviation used to quantify uncertainty.

### 2.3 Alloy Screening Method

#### 2.3.1 Multi‐Objective Layer‐by‐Layer Screening

To satisfy the actual engineering requirements of turbine‐disk applications, this study introduces a stepwise microstructure‐property screening strategy for identifying high‐performance Ni‐based PM superalloys. Candidate alloys were first screened for lightweight performance via mixing rule calculations [29], retaining those lighter than the base alloy. Microstructural screening criteria were then applied to identify alloys with stable microstructures and sufficient γ′ strengthening, specifically those with no TCP phases and *V*_γ′_ >50%. Finally, mechanical property thresholds at 750°C—yield strength >1100 MPa and creep time > 100h at 480 MPa—were enforced to satisfy the practical service requirements of turbine‐disk materials.

#### 2.3.2 Composition Selection Based on Voting Rules

In the vast composition design space, multiple candidate alloys may satisfy the target performance criteria. Then, the Borda count voting rule [30] was applied for accumulating performance scores to select the optimal composition for experimental verification.

## 3 Results

### 3.1 Construction of a Multi‐Source Dataset

A multi‐source dataset was constructed from high‐throughput thermodynamic calculations, experiments, and literature. The HTC dataset comprises composition, phase constituents, *V*_γ′_, *S*_γ′_, and *N*_γ′_ for 6561 alloys, as shown in Figure S2a, with compositions varying from single‐ to multi‐element modifications relative to the base alloy. The calculated alloys cover both γ/γ′‐dominated microstructures and TCP‐containing phase constitutions, where the TCP phases are mainly σ and P. *V*_γ′_ ranges from 45% to 66% (most >50%), *S*_γ′_ below 100 nm, and *N*_γ′_ is 10^20^–10^21^ m^−3^. The HTE dataset was derived from diffusion‐multiple based on alloy 10 after 1000 h aging at 750°C, yielding 302 data points. Within the dataset, there are 231 TCP‐free compositions and 71 TCP‐containing compositions. These stable alloys exhibit a *V*_γ′_ of 41%–55%, *S*_γ′_ exceeding 200 nm, and *N*_γ′_ lower than computational values. In summary, the computational and experimental datasets cover a similar compositional range but differ in the manner of compositional variation. The computational dataset involves simultaneous variations of multiple alloying elements, whereas the experimental dataset is limited to variations of fewer elements. Differences in distributions of TCP phase fraction, *V*_γ′_, *S*_γ′_, and *N*_γ′_ between the two datasets indicate the presence of inaccuracies in thermodynamic calculations.

The published dataset comprises 275 yield strength measurements and 129 creep life data points (at 0.2% strain) for nickel‐based PM superalloys. Yield strength spans 677–1356 MPa at 20–816°C, while creep life covers 2–4559 h at 649–816°C and 240–980 MPa. Creep data are concentrated below 1000 h (65% of entries) and span several orders of magnitude (10–10^3^), showing broad uneven distribution as depicted in Figure S2d. Consequently, a logarithmic transformation was applied in subsequent modeling.

**FIGURE 2:**
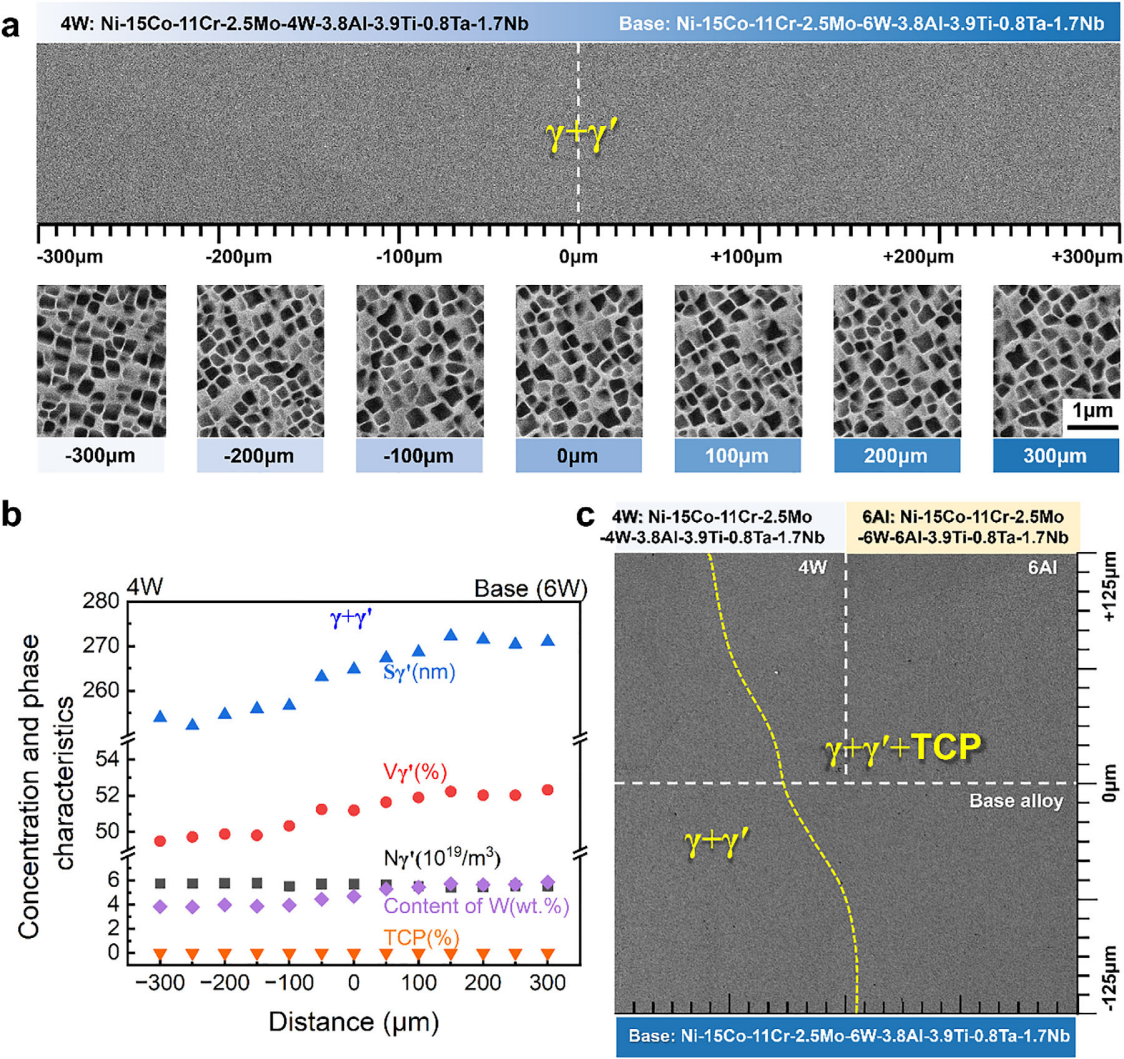
(a) Gradient microstructure in the 4W‐Base diffusion couple after annealing at 750°C for 1000 h, shown at the top, with enlarged images of the region below. (b) Composition distribution and statistics of microstructural changes in the 4W‐Base pseudo‐binary diffusion region. (c) Gradient microstructure in the 4W‐6Al‐Base diffusion couple after annealing at 750°C for 1000 h.

The HTE dataset acquisition is illustrated using 4W‐Base and 4W‐6Al‐Base diffusioncouples (Figure 2). The 4W‐Base pseudo‐binary couple (Figure 2a) exhibits a γ/γ′ two‐phase microstructure, with no TCP phase precipitation within ±300 µm of the initial interface (white dashed line). Figure 2b shows the distribution of W concentration, which increases from 3.8 to 5.87 wt.% from −300 to +300 µm relative to the original interface. The size and volume fraction of the γ′ phase gradually increase, while the number density shows little variation, as shown in Figure 2b. In the 4W‐6Al‐Base pseudo‐ternary region (Figure 2c), higher Al content promotes preferential partitioning into the γ′ phase, thereby enriching refractory elements (Mo, W, and Cr) in the γ matrix and stimulating TCP precipitation (yellow dashed line). This TCP‐forming tendency is suppressed by reducing the W content, as detailed in Figure S4. The corresponding compositional variations and the statistical results of V_TCP_, *V*_γ′_, *S*_γ′_, and *N*_γ′_ are presented in detail in Figure S4. Data from 16 pseudo‐binary and 12 pseudo‐ternary regions produced 302 compositions with TCP content (197 and 105 points, respectively), and for TCP‐free compositions, *V*_γ′_, *S*_γ′_, and *N*_γ′_ were quantified (Figure S4).

### 3.2 Prediction of Microstructural Stability

A phase classification model for TCP precipitation and three regression models for *V*γ′, *S*γ′, and *N*γ′ were established by the TL approach to accurately predict the long‐term microstructural stability of the alloys at 750°C. The HTC dataset served as the source domain, the pseudo‐binary diffusion region data from HTE as the target domain, and the HTE data from pseudo‐ternary diffusion region as the extrapolation testing set. The source domain model provided initial parameters for the target domain model, thereby transferring the learned relationships between alloying elements and microstructural features. During the training of the target domain, the parameters of all pretrained hidden layers except the last one were frozen. After removing the last hidden layer, two new hidden layers with 32 and 16 neurons, respectively, were introduced. Then, the model converged after approximately 1000 epochs. The network architectures before and after the addition of the new layers are summarized in Table S2. The performance of the TCP phase classification model is summarized in Table 1. The accuracies of the source domain and target domain are higher than 94.27%, the recall are higher than 87.50%. The high accuracy and recall values demonstrate that the model can reliably identify the presence or absence of the TCP phase, particularly enabling accurate recognition of TCP‐free alloys and thereby facilitating alloy design. On the extrapolation testing dataset, the TL model attains an accuracy of 90.48% and a recall of 95.06%. These results indicate that, after being calibrated with a limited amount of binary experimental data, the model can achieve accurate predictions on the ternary extrapolation testing dataset.

**TABLE 1:** Accuracy and Recall of the TCP Phase Classification Model.

	Source domain		Target domain		Extrapolation testing Set
	Training set	Testing set	Training set	Testing set	
Accuracy	99.92%	99.71%	94.27%	95.00%	90.48%
Recall	96.77%	87.50%	99.16%	100.00%	95.06%

**FIGURE 3:**
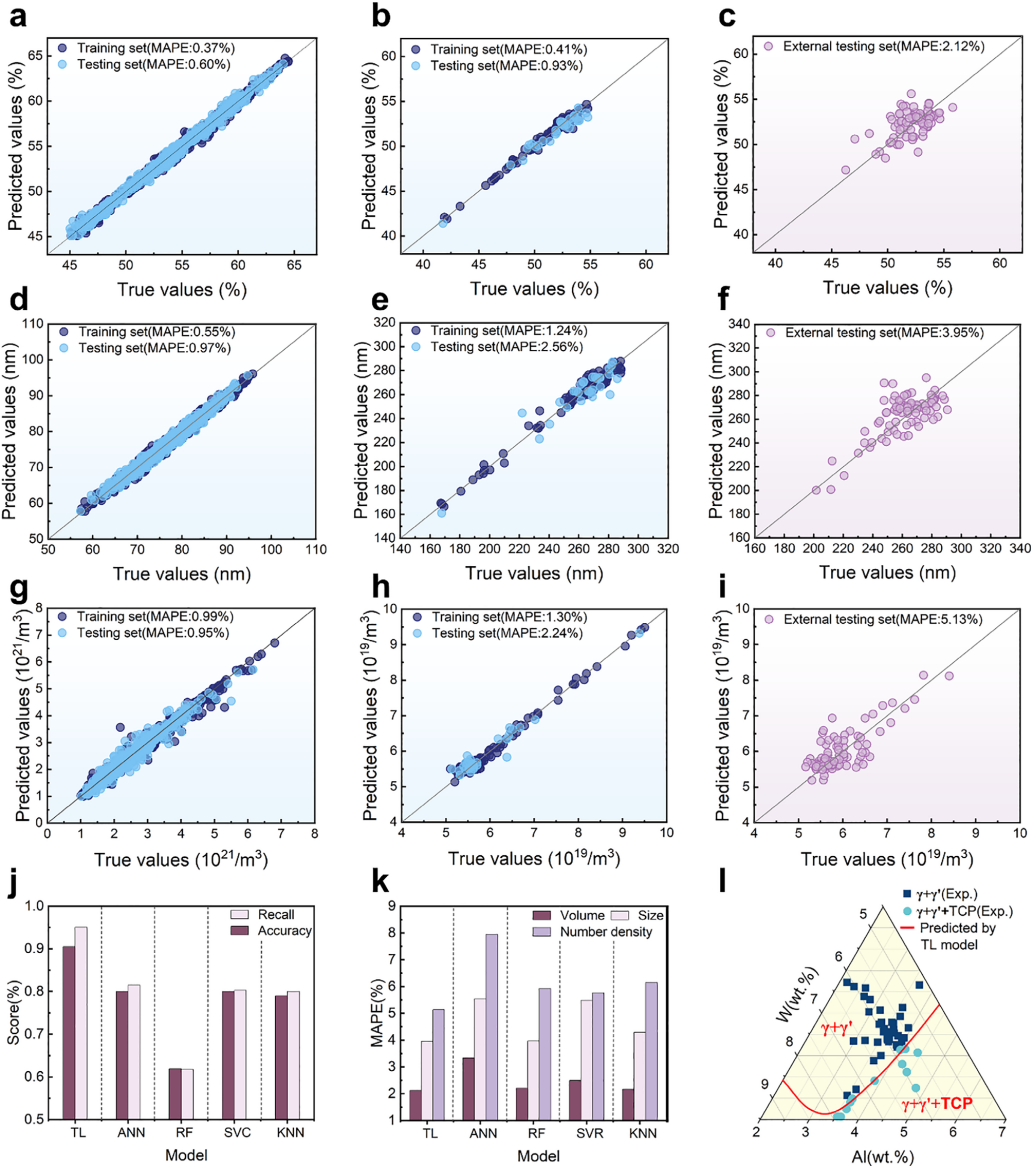
Modeling results of the γ′ phase volume fraction model: (a) source domain, (b) target domain, and (c) extrapolation testing set. γ′ phase size model: (d) source domain, (e) target domain, and (f) extrapolation testing set. γ′ phase number density model: (g) source domain, (h) target domain, and (i) extrapolation testing set. Comparison of prediction accuracy on the extrapolation testing set for the (j) TCP phase classification model and (k) MAPE of γ′ phase features prediction regression model, established using TL and traditional algorithms. The TL model achieves higher accuracy than traditional models. (l) The pseudo‐ternary isothermal section of Base‐xAl‐yW alloys at 750°C, comparing experimental data with TL predictions”.

In alloy design, the target alloys are typically required to maintain a stable γ/γ′ microstructure and remain free of detrimental TCP phases to ensure reliable long‐term service. Therefore, prior to modeling the phase morphological parameters of the γ', all alloy compositions in the HTC dataset were first screened using the TCP phase classification model. Only TCP‐free data were used to establish the TL‐based regression models for *V*_γ'_, *S*_γ',_ and *N*_γ'_. As shown in Figure 3a,b,d,e, most data points lie close to the y = x line, indicating reasonable agreement between the predicted and true values. The three TL‐based models achieve MAPE values below 2.24%. On the extrapolation testing dataset, the TL models achieve MAPE values of 2.21%, 4.28%, and 5.13%, respectively.

We further compared the proposed method with models directly established from experimental data using traditional machine‐learning algorithms. Among the traditional methods, ANN achieved the highest accuracy and recall on the ternary diffusion region, reaching 80.00% and 81.48%, respectively, which are still far lower than the 90.48% and 95.06% achieved by the TL model. Similarly, all γ' phase features TL regression models exhibited lower prediction errors than those built with ANN, SVR, RF, and KNN. More detailed results are shown in Figures S5–S7. Compared with the direct modeling method, the TL model integrates HTC and HTE data, thereby enabling a more accurate capture of the effects of alloying elements on phase features.

Figure 3l shows the isothermal section of the Base‐xAl‐yW alloy at 750°C. The experimental data are relatively sparse, making it difficult to fully and accurately characterize the entire continuous compositional space. Significant discrepancies exist between the thermodynamic calculations and experiments, with a large number of alloys that are experimentally free of TCP phases being incorrectly predicted to contain TCP. The TCP predictions of the TL model are consistent with the experimental results, particularly near the phase boundaries. This approach not only guides alloy composition design by predicting phase constitution and precipitate morphology but can also be applied to phase‐diagram construction, for example, to precisely delineate phase boundaries. This improvement is attributed to the effective transfer of prior knowledge from the source domain, which enables the systematic biases in the computational data to be corrected using only a limited amount of experimental data. The t‐SNE visualization of hidden‐layer output vectors further supports this interpretation. As shown in Figure S9, the frozen layers pretrained on the HTC dataset already exhibit a preliminary ability to distinguish between TCP‐free and TCP‐containing alloys. The newly added hidden layers further sharpen this separation and improve the discriminative ability of the transferred representations, indicating that they help correct the residual bias inherited from the source domain.

### 3.3 Prediction of Short‐Term Mechanical Properties

**FIGURE 4:**
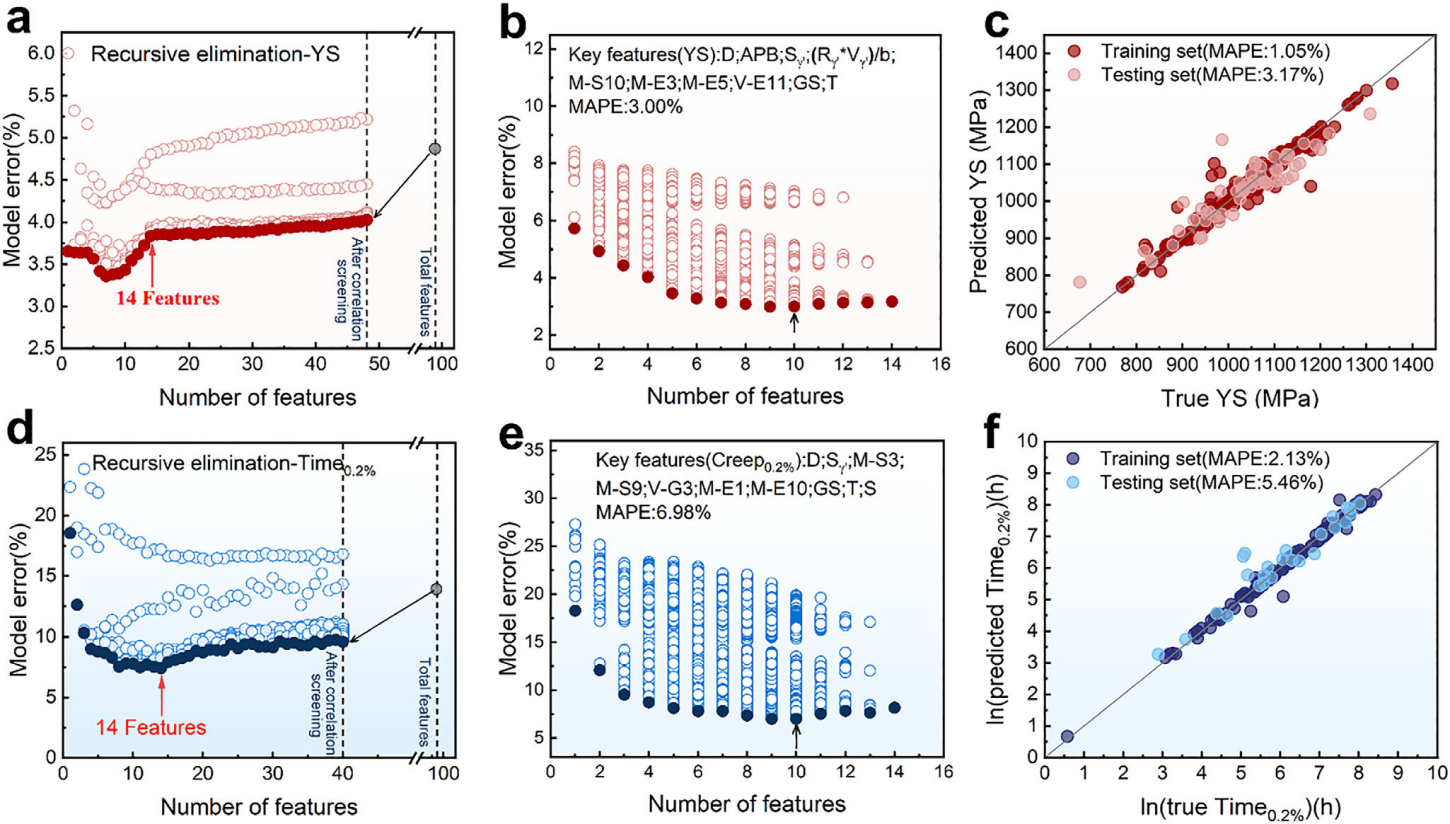
Feature selection process and modeling results for yield strength and the time of 0.2% creep strain. (a,d) Results after the Pearson correlation filtering and recursive feature elimination. (b,e) Exhaustive search results. (c,f) Prediction models.

To enable the machine learning models to capture the effects of alloy composition and microstructure on mechanical properties, Pearson correlation analysis, recursive feature elimination (RFE), and exhaustive feature screening were employed to identify the key descriptors for yield strength and the time of 0.2% creep strain, as shown in Figure 4. First, for each pair of features with a Pearson correlation coefficient greater than 0.9, two separate models were trained using each feature individually as input. The feature associated with the larger model error was then eliminated, while the one yielding better predictive performance was retained. After correlation filtering, 49 and 41 features were used for RFE for yield strength and creep time, respectively. To avoid prematurely discarding potentially informative descriptors due to the greedy nature of RFE, the number of features entering exhaustive screening was determined by balancing computational feasibility with the need to preserve potentially informative feature combinations. For yield strength, the inflection point corresponding to a marked reduction in prediction error was selected (14 features), while for creep time, the minimum‐error point was used (14 features). Finally, both models converged to 10 key features. These features include physical and chemical descriptors, microstructural parameters, and domain‐knowledge‐based features such as γ′ phase size and diffusion coefficient. This demonstrates the necessity of constructing a multivariate feature set.

Figure 4c,f presents the predictive performance of the yield‐strength model and the creep‐time model, respectively. For the yield‐strength model, the MAPE values for the training and test sets are 1.05% and 3.17%, respectively. For the creep‐time model, the corresponding MAPE values are 2.13% and 5.46%. In both cases, the MAPE values on the training and test sets remain below 5.5%, demonstrating that the developed models exhibit strong predictive accuracy and reliability.

### 3.4 Design and Experimental Validation of High‐Strength, Creep‐Resistant Nickel‐Based PM Superalloys

**FIGURE 5:**
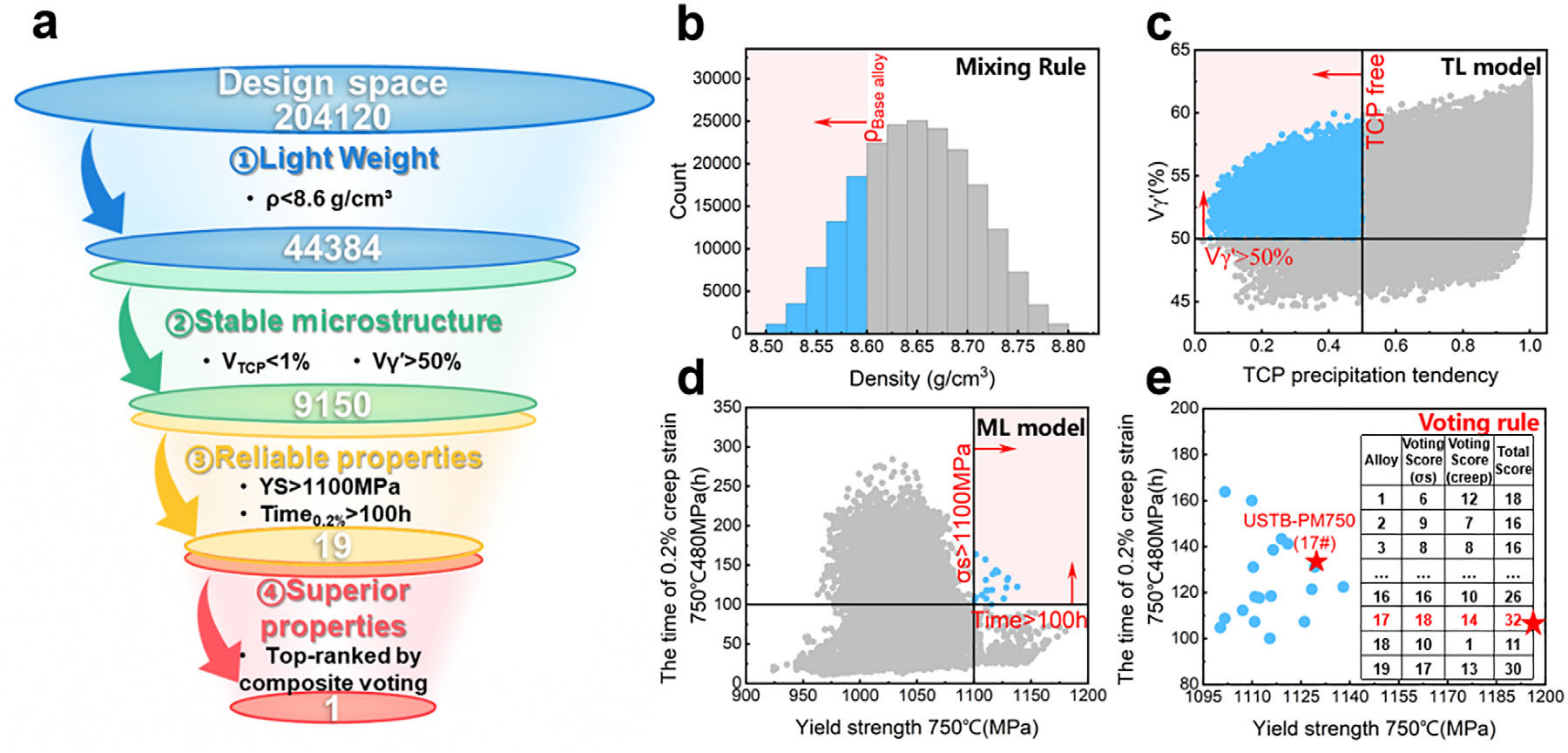
(a) Process of high‐performance alloy selection. (b) Stepwise application of the density criterion (density <8.6 g/cm^3^). (c) Screening of TCP‐free alloys with *V*_γ′_ >50%. (d) Further screening based on yield strength >1100 MPa and the time of 0.2% creep strain >100 h, reducing the initial 204120 candidate compositions to 19. (e) Selection of the optimal alloy from these 19 candidates using a voting rule.

Figure 5 illustrates the selection from 204120 candidate alloys via multi‐objective hierarchical screening. 19 alloys satisfied all criteria for density, long‐term microstructural stability, *V*_γ′_, yield strength, and creep resistance. These were ranked by Borda count voting based on yield strength at 750°C and the time of 0.2% creep strain at 750°C/480 MPa. The composition with the highest score, Ni‐18Co‐11Cr‐8.5 (Mo + W)‐10.4 (Al + Ti + Ta + Nb), is predicted to exhibit a γ′ volume fraction of 50.7%, a yield strength of 1130 MPa, and a creep life of 134 h, with standard deviations of 0.49%, 15 MPa, and 38 h, respectively. Designated USTB‐PM750, this alloy was selected for experimental verification.

**FIGURE 6:**
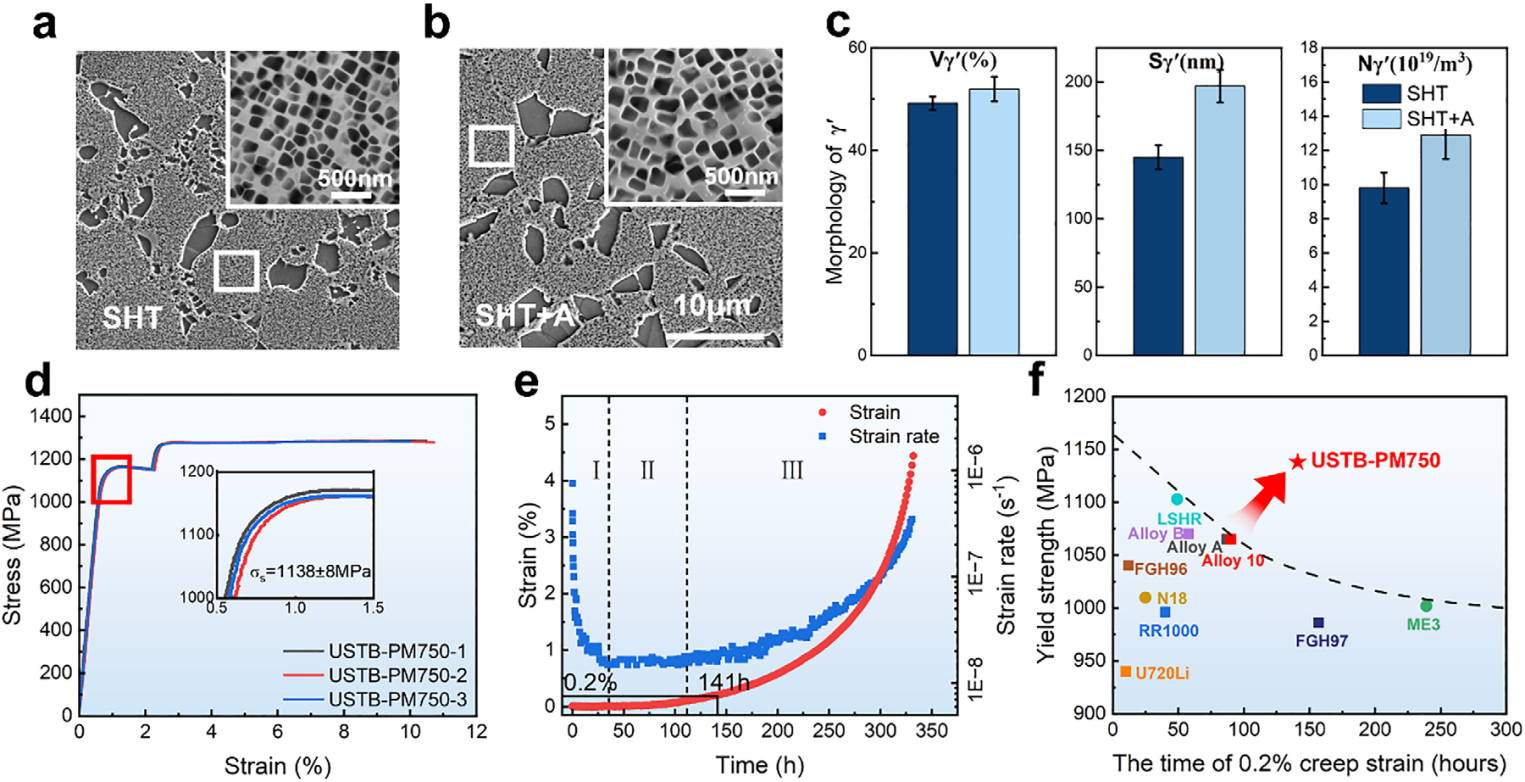
Experimental validation of the designed alloys. (a) Microstructure of the USTB‐PM750 alloy after SHT. (b) Microstructure of the SHT‐treated USTB‐PM750 alloy after aging at 750°C for 1000 h (SHT+A). (c) Statistical analysis of γ′ morphology. (d) Tensile stress–strain curve at 750°C. (e) Creep strain curve at 750°C and 480 MPa. (f) Comparison of the high‐temperature mechanical properties of the USTB‐PM750 alloy with existing nickel‐based PM superalloys [31, 32, 33, 34, 35, 36], where creep properties for alloys marked with circular symbols were extrapolated using the Larson–Miller equation.

After standard heat treatment (SHT), as shown in Figure 6a, USTB‐PM750 exhibits primary γ′ at grain boundaries, secondary γ′ within grains, and γ matrix. The average grain size was 6.76 µm. The alloy demonstrates good microstructural stability, as seen in Figure 6b, with no TCP precipitation after 1000 h of aging at 750°C. The morphology of the secondary γ′ phase after heat treatment is shown in Figure 6c. After long‐term aging, the size and volume fraction of the secondary γ′ phase increase, while its number density decreases slightly. The measured *V*_γ′_ of 51.96% differed by only 1.68% from prediction. The measured density was 8.33, which was 0.26 g/cm^3^ lower than calculated values. As illustrated in Figure 6d, the measured yield strength at 750°C was 1138 MPa, obtained as the average of three repeated measurements. Creep strain curves at 750°C/480 MPa (Figure 6e) indicate that a strain of 0.2% is reached at 141 h, falling within the prediction interval defined by the mean prediction and its associated uncertainty. This indicates that the model provides reasonably accurate predictions within the expected variability, as shown in Table S10. Comparative analysis with existing PM nickel‐based superalloys at 750°C [31, 32, 33, 34, 35, 36] confirms USTB‐PM750's superior overall mechanical properties.

## 4 Discussion

### 4.1 Interpretability of the Short‐Term Mechanical Properties Model

The ML model developed in this study not only achieves high predictive accuracy but also exhibits strong physical interpretability, with its reliability stemming from a deep linkage between key material features and the deformation mechanisms. The SHAP analysis indicates that, apart from the test conditions, knowledge ‐ based features that are strongly related to the deformation mechanisms, such as the diffusion coefficient (D) and the antiphase boundary energy (γ_APB_), have a significant impact on the yield strength and creep behavior, as shown in Figure S10. These features determine the deformation behavior of the alloy by influencing the interaction modes between dislocations and the γ' phase. A higher diffusion coefficient accelerates dislocation motion and grain boundary migration, and because the diffusion coefficient is directly proportional to the creep rate [37], reduces the yield strength and creep life of the alloy. The value of *γ*_APB_ is a direct criterion for determining whether a/2<110>{111} dislocations can penetrate the γ' phase to form antiphase boundaries [2]. A larger *γ*_APB_ corresponds to a higher strength. Electronegativity, ionization energy, electron concentration, and other electronic characteristics are also of high importance. Electronic behavior is the essential manifestation of the mechanical properties of metallic materials. Charge transfer directly affects the strength of atomic bonds [38], the strengthening effect of γ/γ' interfaces [39], and the degree of element segregation at grain boundaries [40]. The size of the γ' phase obtained from the structure prediction model is a key feature for establishing the two models, and it directly determines the degree of the γ/γ' misfit and the manner in which dislocations pass through γ'. In addition, the key feature of the yield strength model, *R*_γ'_^*^ *V*_γ'_ /b (where b is the Burgers vector and *R*_γ′_ = *S*_γ′_/2 is the radius of the γ′), is a knowledge‐based feature constructed from the two structure characteristics of *R*_γ'_ and *V*_γ'_, and it is positively correlated with the increase in yield strength caused by coherent strain strengthening [41].

### 4.2 Analysis of the Performance Enhancement Mechanism in USTB‐PM750

Complex elemental interactions obscure composition‐microstructure‐property relationships in highly alloyed nickel‐based superalloys, yet understanding these linkages is essential for high‐performance alloy design. This study analyzes USTB‐PM750 microstructure before and after deformation to elucidate mechanisms underlying its superior yield strength and creep performance.

**FIGURE 7:**
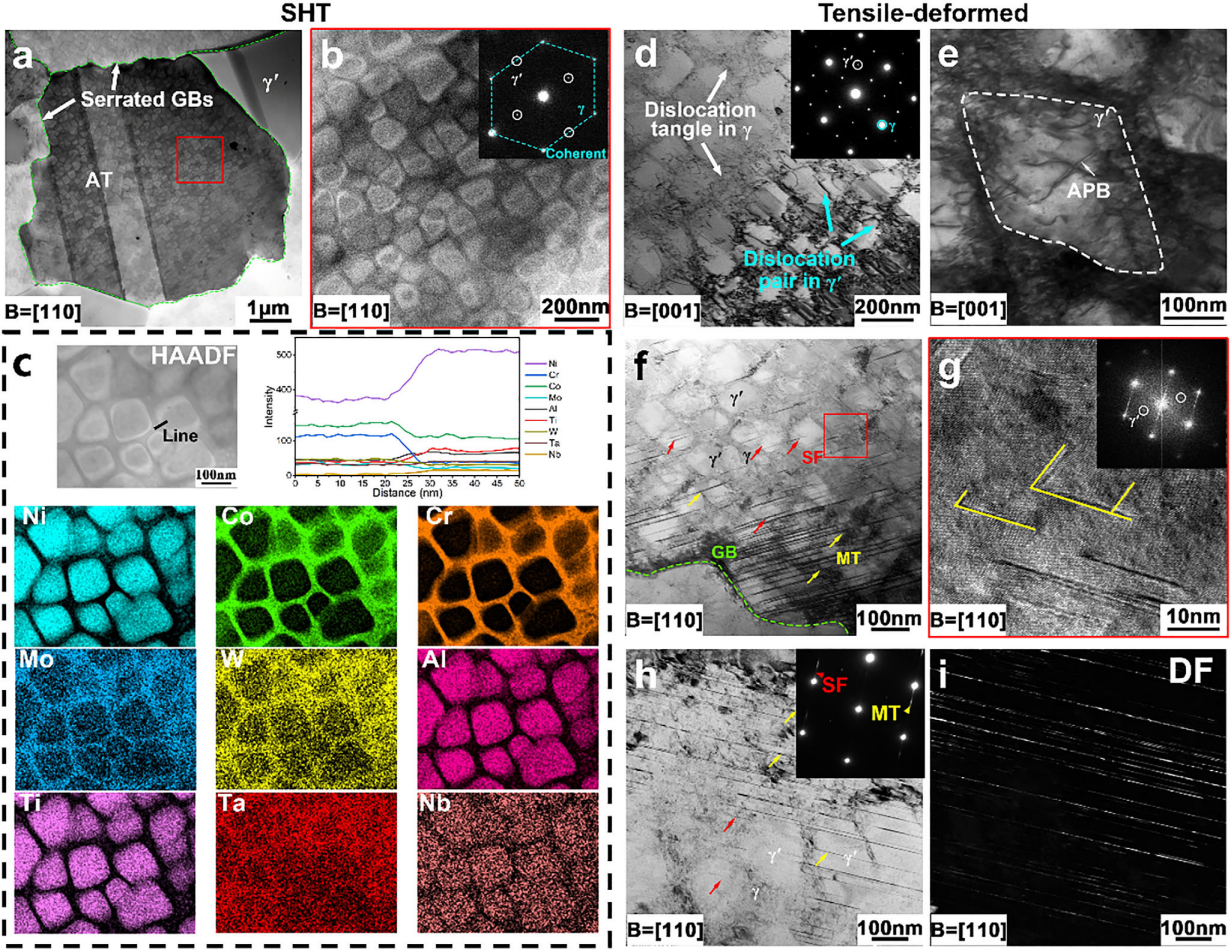
(a) BF image of the USTB‐PM750 alloy after SHT. (b) BF TEM image showing the γ′ phase. (c) STEM‐HAADF image and compositional analysis of the γ′ phase in USTB‐PM750. (d) BF TEM image of the USTB‐PM750 alloy after tensile deformation. (e) STEM‐BF image after tensile deformation, showing APBs formed via dislocation shearing of the γ′ phase. (f) BF TEM image showing SF after tensile deformation. (g) HRTEM image illustrating defect interactions. (h) BF TEM image and (i) DF images of SF and MT traversing the γ and γ′ phases.

As shown in Figure 7, the grain boundaries of the alloy after SHT exhibit a serrated structure, which effectively hinders grain boundary sliding and promotes intra‐grain plastic deformation. The tortuous morphology also significantly delays intergranular crack propagation and extension, thereby improving the high‐temperature performance of the alloy [42, 43]. Additionally, the figure reveals the presence of annealed coherent twins within the grains, the boundary frequency fraction of which was measured by EBSD to be 31% (Figure S11). Based on JMatPro thermodynamic calculations, the stacking fault energy of the USTB‐PM750 alloy is 77.9 J/m^2^, significantly lower than that of Alloy 10 (94.4 J/m^2^). This lower stacking fault energy reduces the critical shear stress for dislocation slip, facilitating the nucleation and growth of twins during annealing, thus forming a high‐density annealed twin structure. These twin boundaries have a low interfacial energy and maintain good thermal stability at high temperatures, contributing to high‐temperature strengthening by hindering dislocation motion [44]. Figure 7c shows the compositional analysis of the γ′ phase after SHT. The results indicate strong enrichment of Ni, Al, Ti, and Nb in the γ′ phase, with Ta showing slight enrichment, while the remaining elements are enriched in the γ matrix. The segregation of large‐sized, slow‐diffusing elements such as Nb and Ta can substitute for Al in the γ′ phase (Ni_3_Al), increasing the *γ*_APB_ and making γ′ more resistant to dislocation shearing during deformation, thereby enhancing the alloy's strength and creep resistance [45].

Bright‐field (BF) images of the tensile‐fractured sample show extensive dislocation glide within the γ channels, where dislocations entangle to form networks and multiple slip systems are activated. Dislocations pile up at the γ/γ′ interfaces, generating stress concentrations, and subsequently enter the γ′ phase in pairs to produce APBs shown in Figure 7e. Meanwhile, after full dislocations dissociate at the phase boundary, the leading partial dislocations enter the γ′ phase and form superlattice stacking faults, as shown in Figure 7f. These stacking faults (SF) exhibit three morphologies: isolated faults within γ′, interacting faults within γ′, and faults traversing both γ and γ′ phases. HRTEM analysis indicates that the isolated faults mainly originate from the dissociation of perfect dislocations at the γ/γ′ interface, producing single intrinsic stacking faults that pin mobile dislocations and contribute to the initial work hardening and strengthening [46]. The interactions between stacking faults within the γ′ phase, as illustrated in Figure 7g, lead to the formation of Lomer–Cottrell (LC) locks on intersecting slip planes, which significantly enhance the strength of the γ′ phase [47, 48]. In addition, some stacking faults that traverse γ and γ′ can thicken during deformation to form microtwins (MT), thereby accommodating plastic deformation. At larger strains, high‐density MTs generated by cooperative partial dislocation slip accommodate plastic deformation. The combined effects of annealed twin boundaries and deformation‐induced stacking faults and micro‐twins thus underpin the alloy's exceptional high‐temperature strength. Collectively, these effects are responsible for the high strength of the alloy.

**FIGURE 8:**
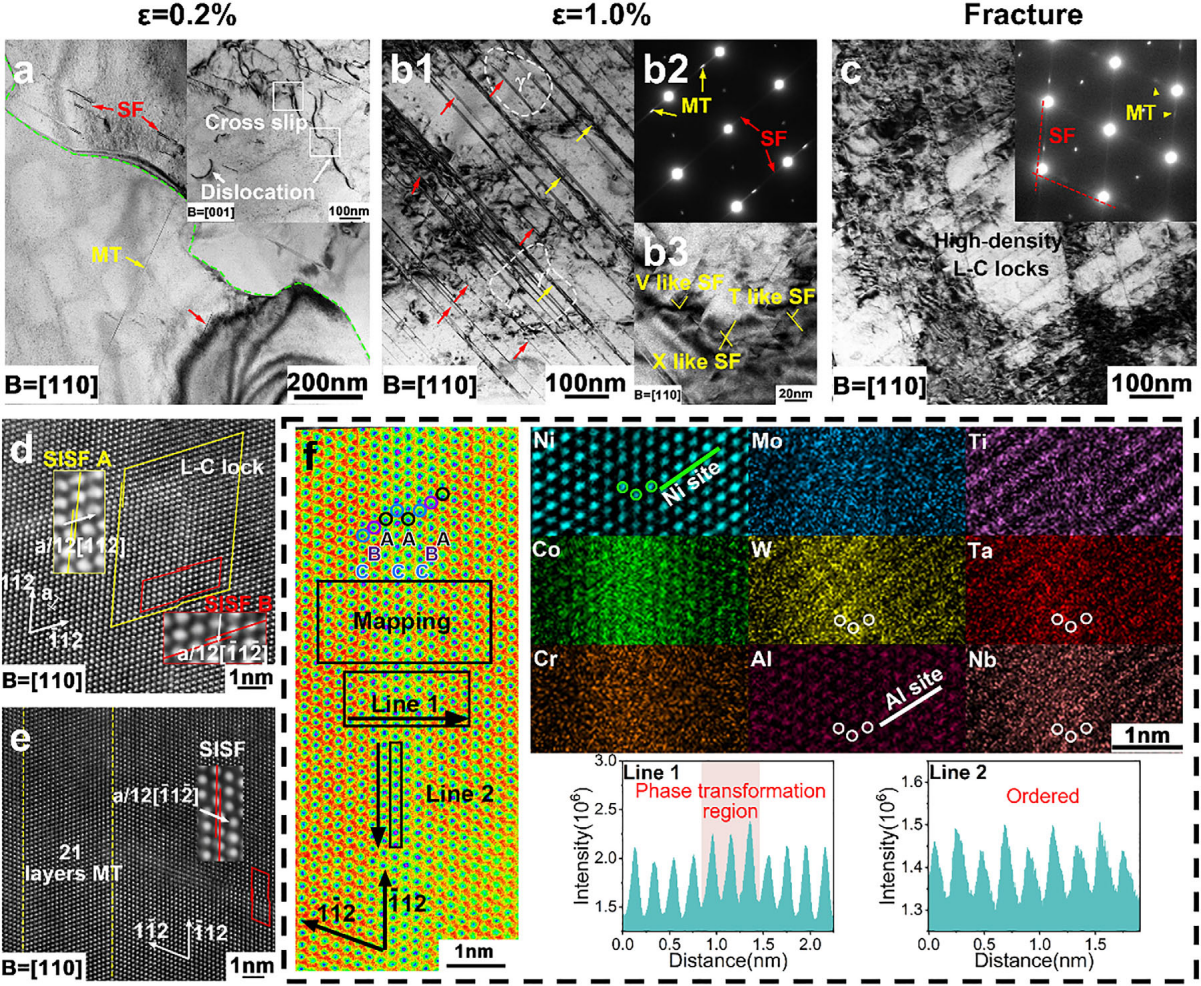
(a) BF images of USTB‐PM750 alloy after creep strain to 0.2% at 750°C/480 MPa. (b1) BF image after creep deformation to 1%. (b2) Diffraction pattern of b1. (b3) BF image at higher magnification showing SF interactions. (c) BF image and its diffraction pattern after creep fracture. (d) HRTEM of the V‐type interaction structure (SISF‐SISF) in the γ′ phase. (e) HRTEM of the T‐type interaction structure (MT‐SISF) in the γ′ phase. (f) STEM‐HAADF image of SISF with atomic‐level EDS elemental mapping and contrast intensity line scan.

The creep deformation mechanism at 750°C/480 MPa was also examined. The microstructure of the alloy before deformation is the same as that in Figure 7a. As shown in Figure 8a, SF and MT form on a single slip system as thin features with dispersed dislocations at 0.2% strain. Some dislocations exhibit nearly 90° bending, indicating cross‐slip and Kear‐Wilsdorf lock formation that enhances creep strength [2]. At 1% strain, as illustrated in Figure 8b1, SF and MT density and thickness increase significantly, becoming the dominant deformation mechanism. Figure 8b3 shows the onset of V‐type, T‐type, and X‐type defect interactions. Near fracture, these features intertwine to form a high‐density LC lock network that hinders dislocation slip and yields a creep rupture life exceeding 300 h. High‐angle annular dark field (DF) scanning transmission electron microscopy (HAADF‐STEM) reveals the lock formation process. As shown in Figure 8d, the V‐type structure forms when a partial dislocation 30°a/6<$\bar{2}\bar{1}$1> creates superlattice intrinsic stacking fault (SISF) A on the (1$\bar{1}$1) plane, cross‐slips to the ($\bar{1}$11) plane, dissociates into 90°a/3<001> and a new partial 30°a/6<$\bar{2}\bar{1}\bar{1}$>, which then forms SISF B to complete the lock. The T‐type structure shown in Figure 8e is formed by an MT with a thickness of 21 atomic layers located on the ($\bar{1}$11) plane and an SISF located on the (1$\bar{1}\bar{1}$) plane. No cross‐slip of a twinning partial has taken place to produce this fault, as the twin surface is flat and the twin thickness remains unchanged. This instead that suggests that it likely nucleated at the twin boundary [49]. The leading partial dislocation is 30° a/6<$\bar{2}\bar{1}\bar{1}$>.

It is noteworthy that significant solute atom segregation is observed at the SF and MT boundaries. As shown in Figure 8f, Co, Cr, Mo, W, Ta, and Nb are enriched at the SISF, while Ni and Al are significantly depleted. The segregated atoms exhibit ordered positioning. The contrast intensity results perpendicular to the stacking fault plane (Figure 8f, Line 1) show a contrast peak of three atomic layers at the SISF and its vicinity. The contrast intensity detected along the stacking fault plane (Figure 8f, Line 2) alternates between strong and weak, indicating that atomic rearrangement at the stacking fault results in a micro‐regional phase transformation, forming a D019‐χ ordered structure [50, 51, 52]. This transformation hinders shear along {111} planes, preventing further thickening and MT formation. The elemental segregation at the superlattice extrinsic stacking fault is detailed in Figure S12. Similarly, the enrichment of heavy atoms such as W, Ta, and Nb at the MT boundaries (Figure S12) effectively reduces the migration rate of the twin boundaries. The segregation behavior of these elements at planar defects plays an important role in stabilizing the defects and delaying high‐temperature softening [53].

Beyond performance optimization, the integrated framework combining high‐throughput thermodynamic calculations, diffusion‐multiple experiments, and transfer learning offers a resource‐efficient alternative to conventional alloy development, aligning closely with the principles of sustainable materials science by minimizing material waste and energy consumption. Traditional superalloy design relies on extensive trial‐and‐error experimentation, consuming substantial energy, raw materials, and time. By screening 10^5^ compositions via CALPHAD‐based high‐throughput calculations and calibrating with sparse experimental data from diffusion‐multiples, our transfer learning approach reduces the experimental burden compared to conventional methods, minimizing material waste and energy consumption associated with high‐temperature processing and long‐term aging. The hierarchical screening strategy further incorporates density as a design criterion, leading to the development of USTB‐PM750 with a density of 8.33 g/cm^3^. This low density provides potential for lightweight turbine disk applications, complementing the alloy's high‐temperature mechanical performance. These aspects illustrate how data‐driven frameworks can improve alloy development efficiency while maintaining high performance, representing a step toward more sustainable practices in materials research.

## 5 Conclusion

This study presents an integrated design strategy combining high‐throughput computation, experimentation, and machine learning for rapid development of high‐performance nickel‐based PM superalloys. The key conclusions are:

1. TL leverages limited high‐fidelity experimental data to correct large‐scale computational datasets, achieving >90% accuracy and recall for microstructure classification and <5.2% error for γ′ volume fraction, size, and number density predictions.
2. A feature set incorporating precipitate morphology, knowledge‐based descriptors, physicochemical attributes, and testing conditions enabled yield strength and creep life models with <5.5% error. SHAP analysis reveals key feature influences, providing both predictive capability and physical interpretability.
3. Multi‐objective hierarchical screening with voting‐based selection identified a low‐density PM superalloy with stable microstructure, high strength, and excellent 750°C creep resistance from 10^5^ candidate compositions. The designed USTB‐PM750 alloy achieved 1138 MPa yield strength and 141 h to 0.2% creep strain at 750°C/480 MPa, surpassing conventional first‐ to third‐generation PM superalloys and demonstrating potential for next‐generation aerospace hot‐section components.
4. Deformation mechanism analysis reveals that USTB‐PM750's superior performance stems from low stacking fault energy promoting SF and MT formation, leading to high‐density LC lock networks and local phase transformations via solute segregation.

## 6 Experimental Method

The composition of diffusion‐multiple was analyzed by JEOL JXA‐8230 electron probe microscopy (EPMA). Combined with the high‐resolution automatic image acquisition system (SEM+ALTAS) and ImageJ image processing software, we statistically analyzed the volume fraction and morphology of precipitated phases in diffusion‐multiple sections.

Experimental verification was carried out on the designed new type of PM alloy: samples were prepared by melting the master alloy, gas atomization powder‐making, hot isostatic pressing, and isothermal forging. And it was treated with oil cooling at 1160°C for 4 h and air cooling at 750°C for 16 h (standard heat treatment). The grain size of the alloy was observed by using Bruker electron backscattering diffraction (EBSD). The heat‐treated samples were aged at 750°C for 1000 h to observe the microstructure stability of the alloy. The morphology of the sample tissue was observed by Zeiss Merlin field emission electron scanning microscope (FEG‐SEM). The specimens after heat treatment were subjected to tensile tests at 750°C in accordance with the GB/T 220.2‐2015 standard. Creep tests were conducted on the heat‐treated specimens in accordance with the GB/T 2039‐2024 standard at 750°C/480 MPa.

## Funding

This work was supported by the National Natural Science Foundation for Distinguished Young Scholars of China (52425409), National Major Science and Technology Projects of China (2024ZD0608100), and Science and Technology on Advanced High Temperature Structural Materials Laboratory (No. 6142903210306).

## Conflicts of Interest

The authors declare no conflicts of interest.

## Supporting information




**Supporting File**: advs75237‐sup‐0001‐SuppMat.docx.

## Data Availability

The data that support the findings of this study are available from the corresponding author upon reasonable request.
