# Supplementary material for: High‐Throughput Data Generation and Transfer Learning Enabled Microstructure‐Property Integrated Design of Nickel‐Based Powder Metallurgy Superalloy
Source: Adv Sci (Weinh). 2026 Apr 15:e24365. Online ahead of print. doi: 10.1002/advs.202524365 (PMC13334990; doi:10.1002/advs.202524365)
Supplement: Supplementary file 1 — Supporting File: advs75237‐sup‐0001‐SuppMat.docx. [file ADVS-9999-e24365-s001.docx]

Supporting Information

High-Throughput Data Generation and Transfer Learning Enabled Microstructure-Property Integrated Design of Nickel-Based Powder Metallurgy Superalloy

Zixin Li, Hongtao Zhang, Zichao Peng, Yuheng Zhang, Qian Lv, Yihuan Cao, Xuqing Wang, Huadong Fu*, Jianxin Xie

**Alloy Composition Design Space**

Based on the third-generation nickel-based powder metallurgy (PM) superalloy Alloy 10 (Ni-15Co-11Cr-6W-2.5Mo-3.8Al-3.9Ti-0.8Ta-1.7Nb), a comprehensive compositional space was constructed by defining individual ranges and step sizes for 8 elements. This systematic variation resulted in a total of 204120 alloy compositions, as detailed in Table S1.

**Table S1.** The composition design space of nickel-based PM superalloys.

|  | Ni  (wt.%) | Co  (wt.%) | Cr  (wt.%) | W  (wt.%) | Mo  (wt.%) | Al  (wt.%) | Ti  (wt.%) | Ta  (wt.%) | Nb  (wt.%) |
| --- | --- | --- | --- | --- | --- | --- | --- | --- | --- |
| Design space | Bal. | 12.0  15.0  18.0 | 10.0  11.0  12.0 | 5.0  5.5  6.0  6.5  7.0 | 2.5  3.0  3.5 | 3.2  3.4  3.6  3.8  4.0  4.2 | 3.1  3.3  3.5  3.7  3.9  4.1  4.3 | 0.4  0.6  0.8  1.0  1.2  1.5 | 1.3  1.5  1.7  1.9  2.1  2.3 |

The high-throughput computational (HTC) dataset was generated using Thermo-Calc with the equilibrium and TC-PRISMA modules, together with the TCNI12 and MOBNI6 databases. For each element, select three composition points for thermodynamic calculations. Phase constitution and γ′ volume fraction (*V*_γ′_), size (*S*_γ′_), and number density (*N*_γ′_) at 750 °C were calculated via Python scripts using the TC-Python interface. For each alloying element, thermodynamic calculations were performed at only three representative levels: the lower bound and upper bound of its compositional range, and the nominal content in the base alloy.

**Details of Diffusion-multiple**

Based on the selected alloying elements and design space, composition gradients were designed by rationally arranging the diffusion-multiple according to elemental characteristics, yielding the diffusion-multiple configuration shown in Figure S1. The base alloy block measures 24 ×4 ×10 mm, and all other blocks are 3 × 5 × 10 mm. Within the diffusion zones, the composition was analyzed at 100 μm intervals using a JEOL JXA-8230 electron probe microanalyzer (EPMA) with a 5 μm beam diameter. In combination with a high resolution automated image acquisition system (SEM + ATLAS) and ImageJ software, the volume fraction and morphology of the precipitated phases at these composition points were quantitatively analyzed. For each diffusion couple, alloy compositions were measured along and perpendicular to the initial interface using a beam spot with a diameter of 5 μm, and each measured spot was defined as an individual alloy composition.


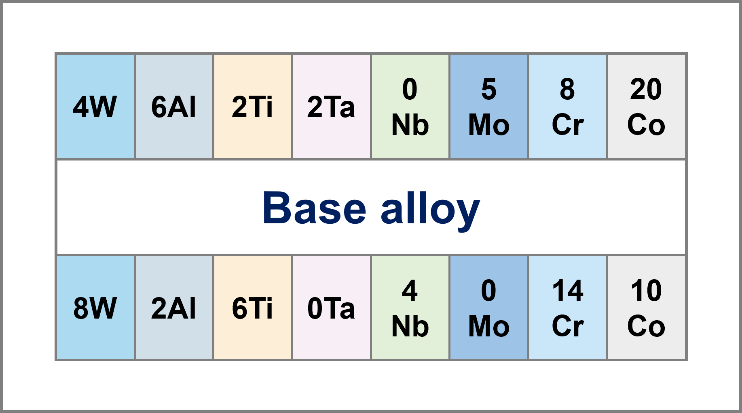


**Figure S1.** Schematic diagram of the diffused-multiple.

**Quantification of precipitate characteristics**

*V*_γ′_, *S*_γ′_ and *N*_γ′_ represent the volume fraction, size and number density of the γ′ phase.. Specifically, *V*_γ′_ was obtained by measuring the area fraction of γ′ precipitates in 5 × 5 μm SEM images at each EPMA sampling point using ImageJ, and *S*_γ′_ was calculated as the average equivalent circle diameter of the γ′ precipitates. *N*γ′ was determined using the following equation:

$N_{\gamma'}=n/V$ (S1)

where *n* is the number of precipitates and *V* is the volume.

For the diffusion multiple, SEM images were taken at each EPMA sampling point; for the newly designed alloys with homogeneous compositions, images were taken at three randomly selected locations on sample.

**Training details of transfer learning (TL) models**

In the TCP phase classification model, a fully connected neural network with an architecture of 64×64×32×16 was first trained on high-throughput thermodynamic calculation data, taking the concentrations of all elements except Ni as input and whether TCP phases precipitate as output. The initial learning rate was set to 1×10^-^³. Then, we froze all hidden layers except the last, added one more hidden layer, retrained the model on the HTE dataset. The parameters are summarized in Table S2. The *V*_γ′_, *S*_γ′_ and *N*_γ′_ models were trained using the same procedure, and parameters are also listed in Table S3.

**Table S2.** TL model parameters. Layers marked with * denote newly added layers. In the target models, the original final hidden layer of the source domain network was removed and replaced with two newly added layers. This modification results in a net increase of one hidden layer in terms of overall network depth.

| Model | Source domains | | | Target domains | | |
| --- | --- | --- | --- | --- | --- | --- |
|  | Learning rate | Hidden layer | Batch size | Learning rate | Hidden layer | Batch size |
| TCP | 1×10^-3^ | (64, 64, 32, 16) | 256 | 1×10^-4^ | (64, 64, 32, 32*, 16*) | 32 |
| *V*_γ′_ | 1×10^-3^ | (128, 64, 32) | 256 | 1×10^-3^ | (128, 64, 32*, 16*) | 32 |
| *S*_γ′_ | 1×10^-3^ | (128, 64, 32) | 256 | 1×10^-3^ | (128, 64, 32*, 16*) | 32 |
| *N*_γ′_ | 1×10^-3^ | (128, 64, 32) | 256 | 5×10^-3^ | (128, 64, 32*, 16*) | 32 |

**Feature set**

The multivariate feature set used for constructing the property prediction models in this work is summarized in **Table S3**.

**Table S3.** Feature set.

| Category | Feature name | Description | Calculation method/source |
| --- | --- | --- | --- |
| Microstructural Features | *V*_γ′_  *S*_γ′_  *N*_γ′_  *w*  Microstructure 1  Microstructure 2  Microstructure 3  Microstructure 4  *GS* | Volume fraction of γ′  Size of γ′  Number density of γ′  γ channel width  1- *V*_γ′_  *V*_γ′_^1/3^  *V*_γ′_^1/3^-1  (*S*_γ′_* *V*_γ′_)/2b  Grain size | γ′ phase features calculated using the TC-PRISMA module based on alloy composition and heat-treatment conditions; grain size obtained from the literature. |
| Domain Knowledge Features | *D*  *Q*  *D0*  *γ*_APB_  $\sigma_{s}$  $\sigma_{p}$  *M*creep  *Md* | Diffusion coefficient  Activation energy  Pre-exponential factor  Antiphase Boundary energy  Solid solution strengthening  Precipitation shearing stress  Index for creep resistance  Md values | [1]  [1]  [1]  [2]  [3]  [4]  [5]  [2] |
| Physical and chemical features | *S1*  *S2*  *S3*  *S4*  *S5*  *S6*  *S7*  *S8*  *S9*  *S10*  *S11*  *S12*  *S13*  *G1*  *G2*  *G3*  *G4*  *G5*  *C1*  *C2*  *C3*  *C4*  *C5*  *C6*  *C7*  *C8*  *C9*  *C10*  *C11*  *C12*  *E1*  *E2*  *E3* | Radii pseudo-potential (Zunger)  Radii ionic (Yagoda)  Radii covalent  Radii metal (Waber)  Distance valence electron (Schubert)  Distance core electron (Schubert)  Volume atom (Villars, Daams)  V^2/3^ Miedema  Atomic environment number (Villars, Daams)  Lattice Constants a  Lattice Constants b  Lattice Constants c  Radii atomic (coordination number 12) (pm)  Group number  Valence electron number  Valence electron number of s  Valence electron number of p  Valence electron number of d  Temperature melting  Temperature boiling  Enthalpy vaporization  Enthalpy melting  Enthalpy atomization  Enthalpy surface Miedema  Enthalpy vacancies Miedema  Energy cohesive Brewer  Modulus compression  Modulus bulk  Modulus rigidity  Modulus Young  Electronegativity (Martynov&Batsanov)  Electronegativity (Pauling)  Electronegativity (Alfred-Rochow) | Elemental physicochemical descriptors were collected from databases and handbooks. For each alloy, the composition-weighted mean and variance of these descriptors were then calculated.  We use $f_{ij}$, where i and j label a given feature and a certain element, respectively, to denote the element features, and $a_{j}$ to represent the content of an element. We then compute the mean fmi and variance fvi for each alloy for features according to following equation, $f_{mi}=\sum\left(f_{ij}a_{j}\right)/\sum Sa_{j}$ $f_{vi}=\frac{\sum\left[\left(f_{ij}-f_{mi}\right)^{2}a_{j}\right]}{\sum Sa_{j}}$    |
| Testing Conditions | *S*  *T* | Stress  Temperature | The data were obtained from the literature. |

**Multi-source dataset**

Published data from the literature on Ni-based PM superalloys (compositions, yield strengths, and times to 0.2% creep strain) were collected using a relatively strict screening and preprocessing workflow, including data completeness evaluation, source reliability assessment, data fusion, and statistical quality control for abnormal-data removal.

The data collection and preprocessing workflow is as follows:

(1) Data completeness evaluation. Each valid data entry was required to include the alloy composition, heat-treatment conditions, testing conditions, microstructural characteristics, and mechanical properties. Entries missing any of these key items were excluded from collection.

(2) Source reliability scoring. Source reliability was assessed using auxiliary criteria such as publication year, article type, and source background, which were used to assign a relative reliability score to each dataset.

(3) Data fusion. When identical compositions with the same processing parameters were reported by multiple sources, the reported values were merged using a reliability-weighted average in order to reduce the influence of single-source bias.

The data distribution of the multi-source dataset is shown in the figure S2.

**
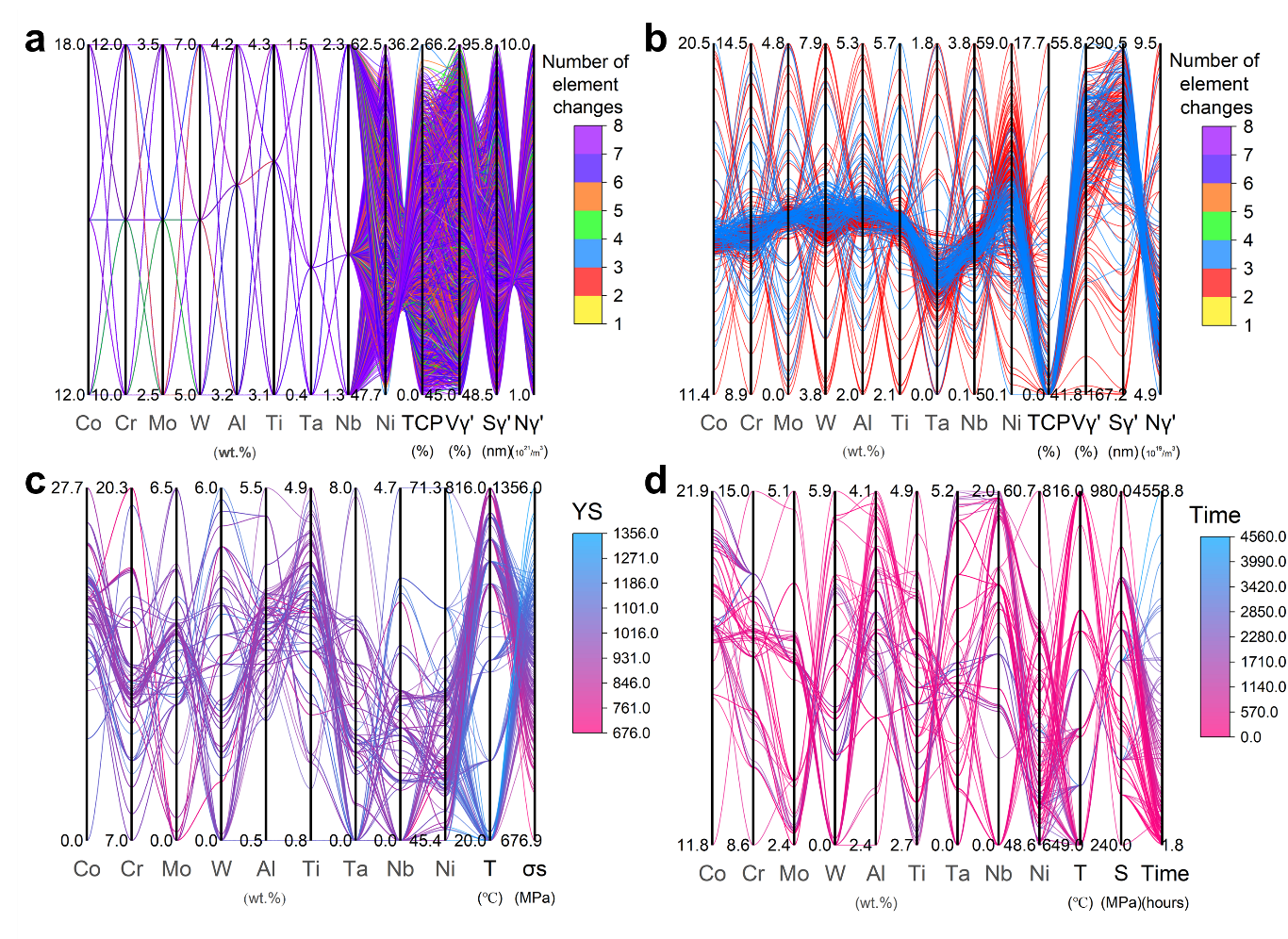
**

**Figure S2.** Distribution of multi-source data. a) HTC data. b) High-throughput experimental (HTE) data. c) Published dataset (yield strength). d) Published dataset (the time of 0.2% creep strain).

The domain shift between HTC and HTE data stems primarily from systematic biases in thermodynamic calculations.


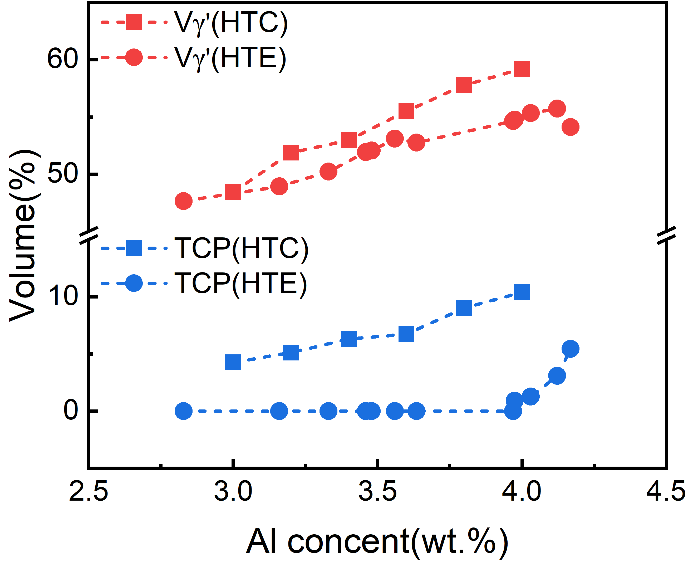


**Figure S3.** Systematic bias in the HTC data. Comparison between thermodynamic calculations and experimental measurements for the effects of elemental variations on TCP phase fraction and γ′ volume fraction.

**Compositional and microstructural characterization of diffusion-multiple**

Within the diffusion-multiple, a total of 16 pseudo-binary diffusion zones (4W–Base, Base–8W, 2Al–Base, Base–6Al, 2Ti–Base, Base–6Ti, 0Ta–Base, Base–2Ta, 0Nb–Base, Base–4Nb, 0Mo–Base, Base–5Mo, 8Cr–Base, Base–14Cr, 10Co–Base, Base–20Co) and 12 pseudo-ternary diffusion zones (4W–6Al–Base, 8W–2Al–Base, 6Al–2Ti–Base, 2Al–6Ti–Base, 0Nb–5Mo–Base, 4Nb–0Mo–Base, 5Mo–8Cr–Base, 0Mo–14Cr–Base, 8Cr–20Co–Base, 14Cr–10Co–Base) were effectively characterized.

Figure S4 shows the microstructural and compositional evolution in the 4W-6Al-Base pseudo-ternary diffusion region. As shown in Figure S4a, the diffusion zone contains γ + γ′ and γ + γ′ + TCP regions, separated by the TCP front marked by the yellow dashed line. Figure S4b provides enlarged micrographs from selected locations in Figure S4a, revealing that the TCP phase exhibits various morphologies, including needle-like, blocky, and rod-like shapes. Figures S4c and S4d present the composition distribution and the statistical analysis of microstructural changes, respectively.

**
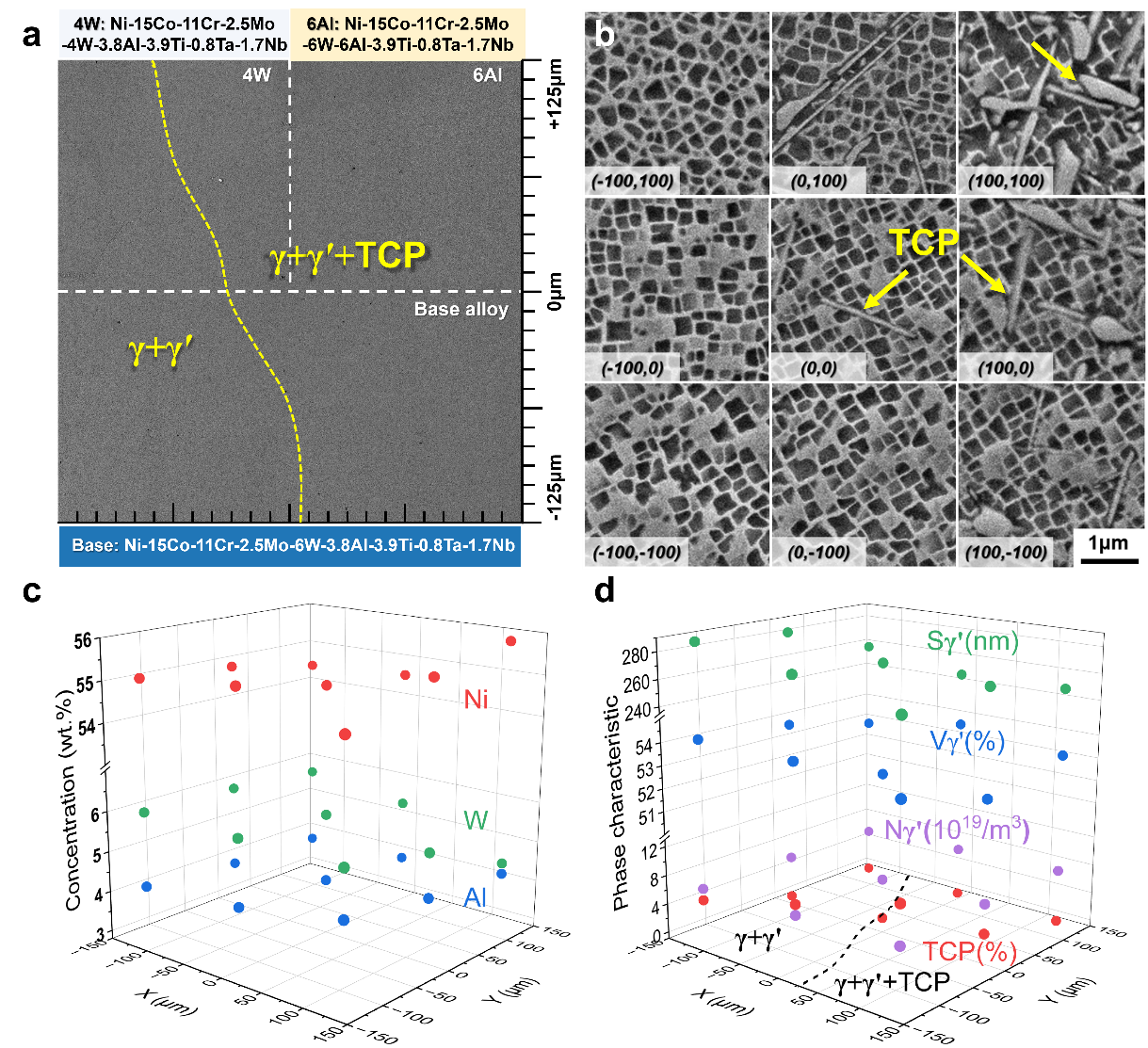
**

**Figure S4.** a) Gradient microstructure in the 4W-6Al-Base diffusion couple after annealing at 750 °C for 1000 h, shown on the left. b) Enlarged images of the region on the right. c) Composition distribution. d) Statistics of microstructural changes in the 4W-6Al-Base pseudo-ternary diffusion region.

Effectiveness of the "High-Throughput Computation + High-Throughput Experiment + Transfer Learning" Approach

Hyperparameters optimization was conducted for all the Artificial neural networks (ANN), support vector machine (SVM), random forests (RF), and k-nearest neighbors (KNN) models in this work. All models were based on the binary-region data in the HTE dataset t and used the same training/test split as that adopted for the TL model. The ANN model employed the same Bayesian optimization method (Optuna) as the TL model for hyperparameters optimization. And the hyperparameters of the RF, SVM, and KNN models were optimized by grid search combined with 10-fold cross-validation. These procedures were intended to ensure a fair comparison among different models and the reliability of the results. The final hyperparameter values used for all models are summarized in Table S4.

**Table S4.** Hyperparameter values for microstructural stability, *V*_γ′_, *S*_γ′_ and *N*_γ′_ models developed using conventional machine learning algorithms.

| Model | ANN | RF | SVM | KNN |
| --- | --- | --- | --- | --- |
| TCP | 8×64×32×16×1  Batch size=32  Learning rate=1×10^-3^ | n_estimators=100  max_depth=5  min_samples_split=5 | C=10  kernel='rbf'  gamma=0.1 | n_neighbors=5  weights='distance'  p=2 |
| Vγ′ | 8×128×64×16×1  Batch size=32  Learning rate=1×10^-2^ | n_estimators=80  max_depth=8  min_samples_split=4 | C=10  kernel='rbf'  gamma=0.1 | n_neighbors=4  weights='distance'  p=2 |
| Sγ′ | 8×64×32×16×1  Batch size=32  Learning rate=1×10^-3^ | n_estimators=100  max_depth=5  min_samples_split=5 | C=2000  kernel='rbf'  gamma=0.1 | n_neighbors=5  weights='distance'  p=2 |
| Nγ′ | 8×64×32×1  Batch size=32  Learning rate=1×10^-2^ | n_estimators=100  max_depth=10  min_samples_split=3 | C=5  kernel='rbf'  gamma=0.1 | n_neighbors=5  weights='distance'  p=2 |

Table S5 summarizes the confusion-matrix statistics of the TCP-phase classification models on the extrapolation testing set, including TP, FN, FP, TN, and the derived F1 score. These results provide a more complete evaluation of the classification performance and further demonstrate the advantage of the TL model over the conventional machine-learning models.

**Table S5.** Confusion-matrix-derived classification metrics for the extrapolation testing set.

| Model | TP | FN | FP | TN | Accuracvy | Recall | Precision | F1 |
| --- | --- | --- | --- | --- | --- | --- | --- | --- |
| TL | 77 | 4 | 6 | 18 | 90.48% | 95.06 | 92.77% | 93.90% |
| ANN | 66 | 15 | 6 | 18 | 80.00% | 81.48% | 91.67% | 87.27% |
| SVC | 65 | 16 | 5 | 19 | 80.00% | 80.25% | 92.86% | 86.09% |
| KNN | 65 | 16 | 6 | 18 | 79.05% | 80.25% | 91.55% | 85.53% |
| RF | 50 | 31 | 9 | 15 | 61.90% | 61.73% | 84.75% | 71.43% |

Figure S5, Figure S6 and Figure S7 compare the extrapolation accuracy of three γ′ features regression models built with TL and with conventional algorithms. Overall, the TL models achieve higher prediction accuracy on the extrapolation test set. And some traditional models even tend to predict different compositions as nearly the same value. This indicates that TL, by leveraging pre-trained knowledge, enhances model generalization and provides a more reliable route for high-throughput prediction of γ′ phase microstructural features.


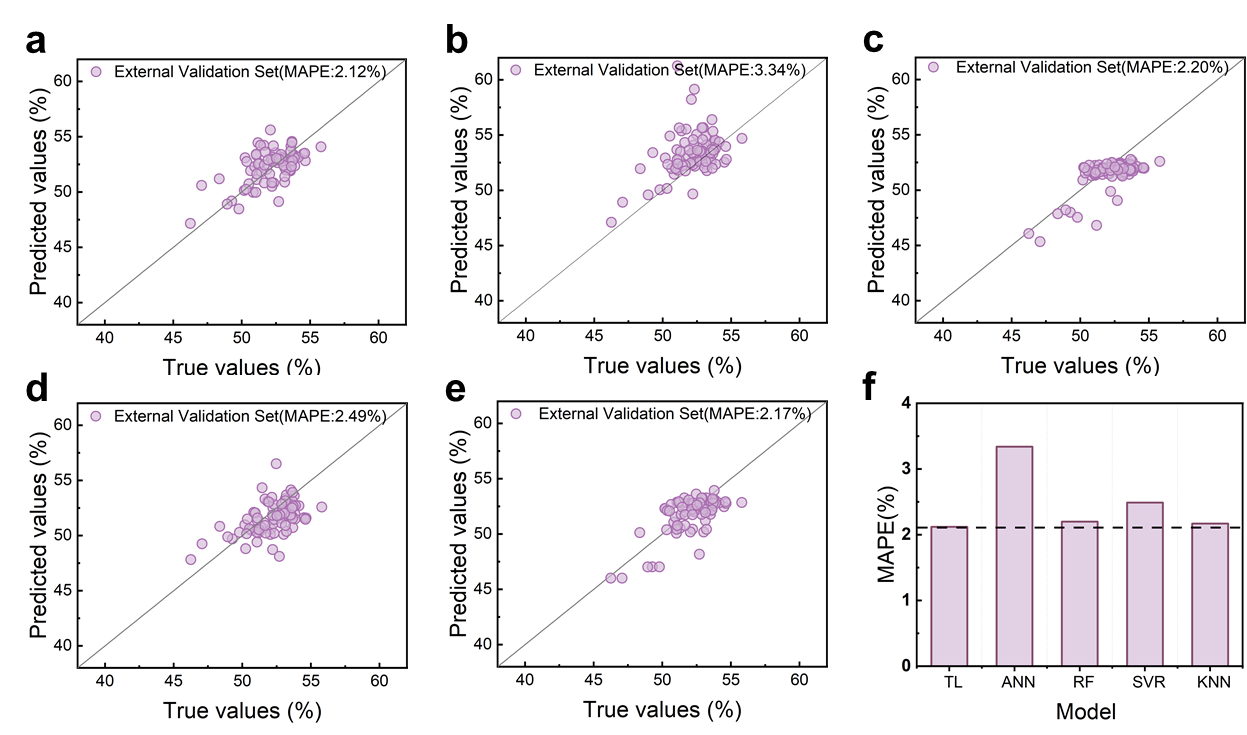


**Figure S5.** Comparison of prediction accuracy on the extrapolation test set for *V*_γ′_ predicted models constructed using a) TL, b) ANN, c) RF, d) support vector regression (SVR), and e) KNN.


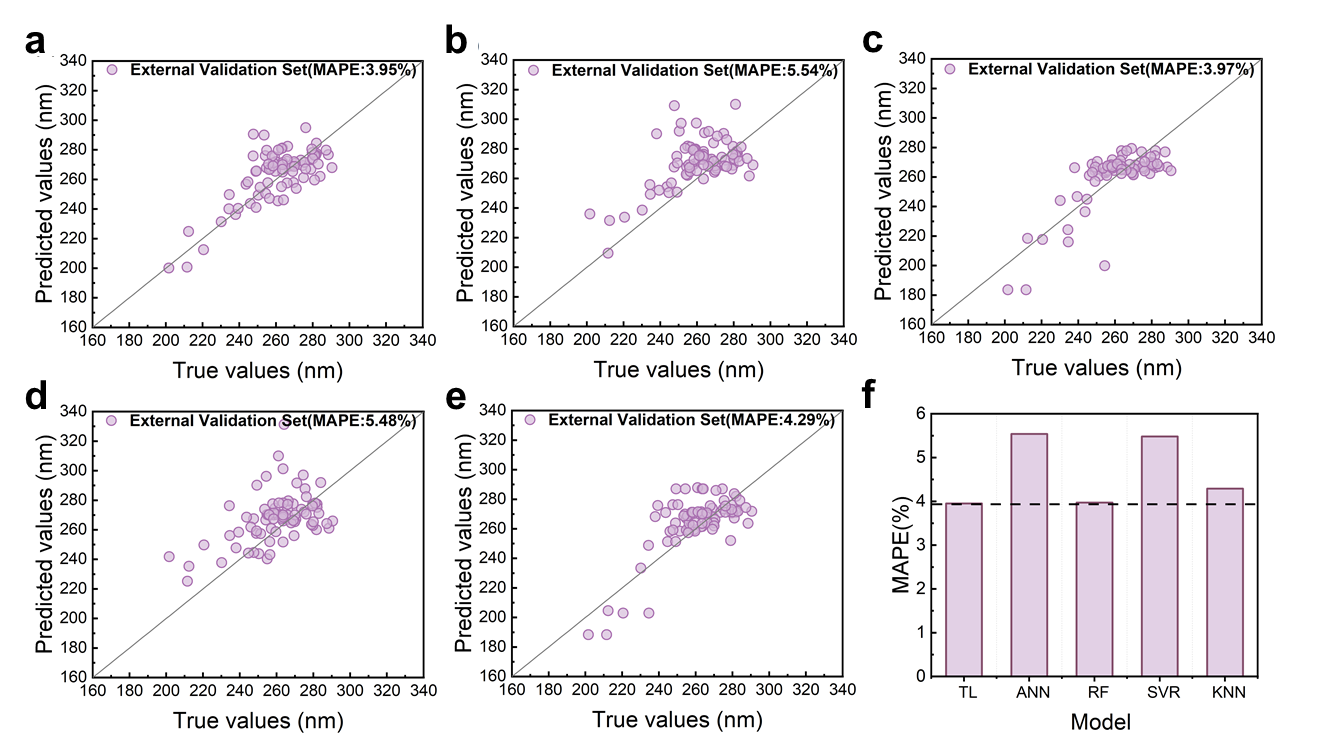


**Figure S6.** Comparison of prediction accuracy on the extrapolation test set for *S*_γ′_ predicted models constructed using a) TL, b) ANN, c) RF, d) SVR, and e) KNN.


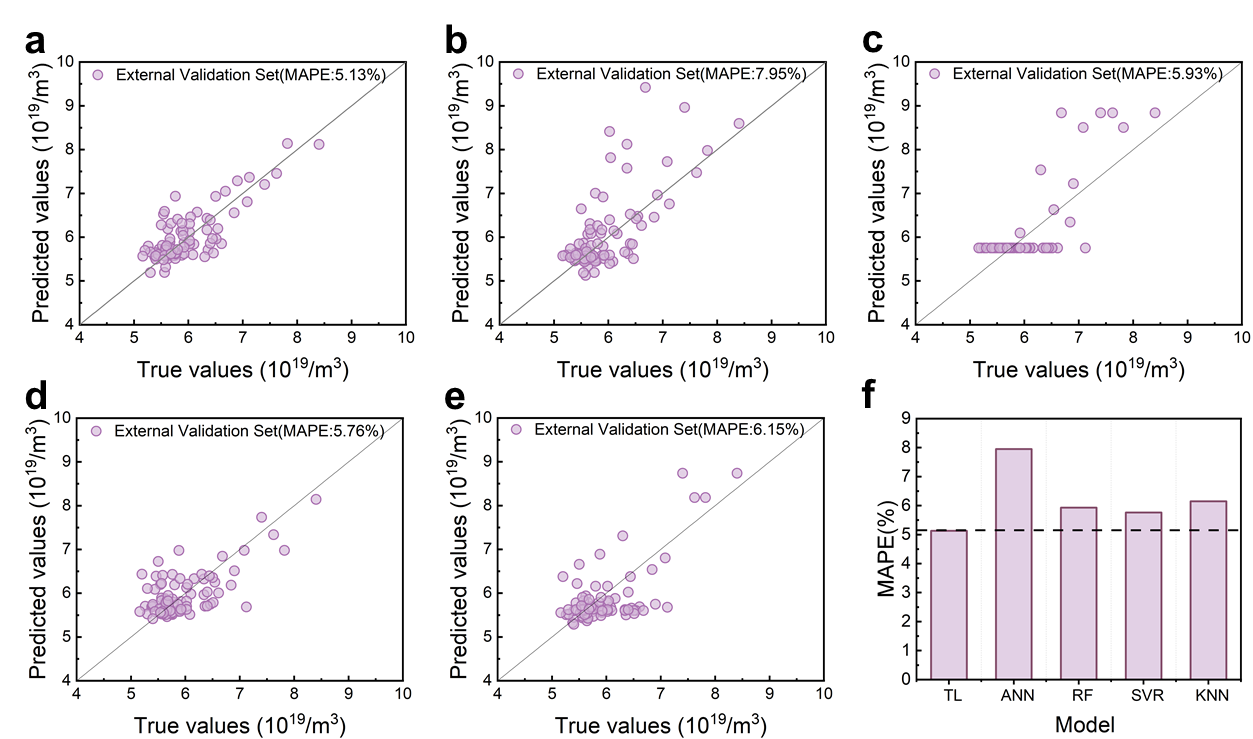


**Figure S7.** Comparison of prediction accuracy on the extrapolation test set for *N*_γ′_ predicted models constructed using a) TL, b) ANN, c) RF, d) SVR, and e) KNN.

To comprehensively evaluate the proposed transfer learning strategy, we conducted a multi-faceted analysis, including model performance assessment, visualization of hidden-layer features, and comparison with alternative approaches.

As shown in Figure S8a and b, the pseudo-ternary isothermal section obtained from thermodynamic calculations exhibits noticeable deviations from the experimental results. In contrast, the TL model accurately captures the phase boundary positions and shows good agreement with experimental observations, especially in the boundary regions. A similar trend is observed for the γ′-related features. As shown in Figure S8c and d, the γ′ phase characteristics predicted by the TL model are consistent with experimental characterizations. These results indicate that the model can effectively learn the underlying knowledge in the computational data and achieve accurate phase prediction.


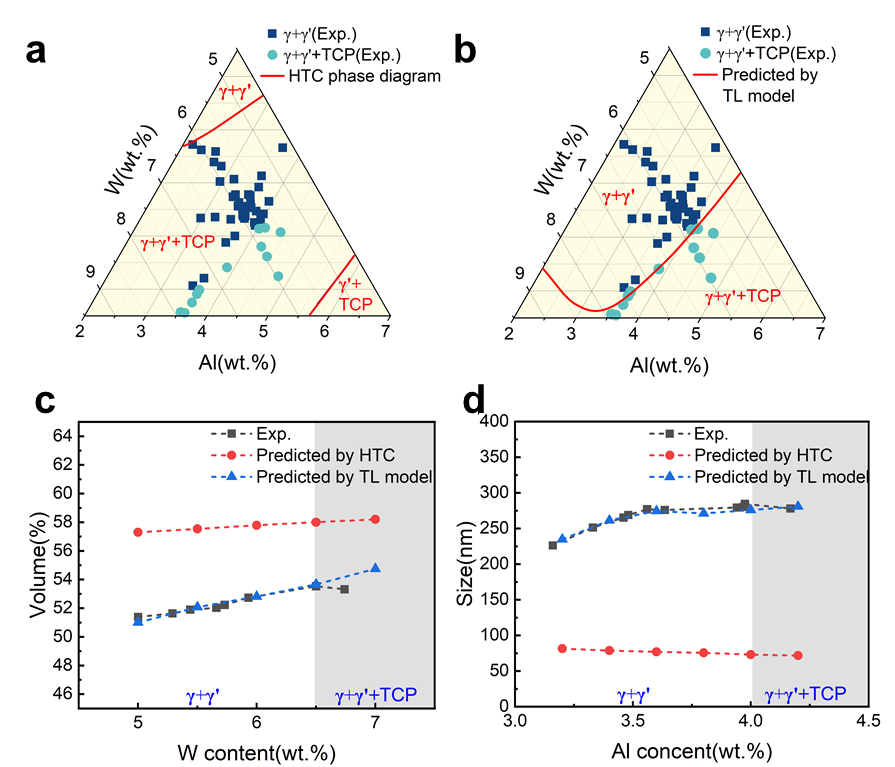


**Figure S8.** The pseudo-ternary isothermal section of Base-xAl-yW alloys at 750℃, comparing experimental data with a) high-throughput thermodynamic calculations and b) TL predictions. c) *V*_γ′_ of Base-yW alloys at 750°C and d) *S*_γ′_ of Base-xAl alloys at 750°C. showing the differences between experimental values and the results from thermodynamic calculations and TL model predictions.

To further examine whether the frozen part of the pretrained network had already captured transferable information, we visualized the hidden-layer outputs of the target domain model using t-SNE. The results show that the outputs from the frozen layers already exhibit a discernible tendency to separate TCP-free and TCP-containing alloys, although the class boundary remains relatively diffuse. Since the subsequent calibration is mainly intended to correct biases in the representation of specific alloying-element effects, it is sufficient to adjust only several layers in the upper part of the network that are more directly related to the output, rather than progressively unfreezing the front layers to relearn the complex composition-microstructure relationships that have already been adequately captured in the source domain.


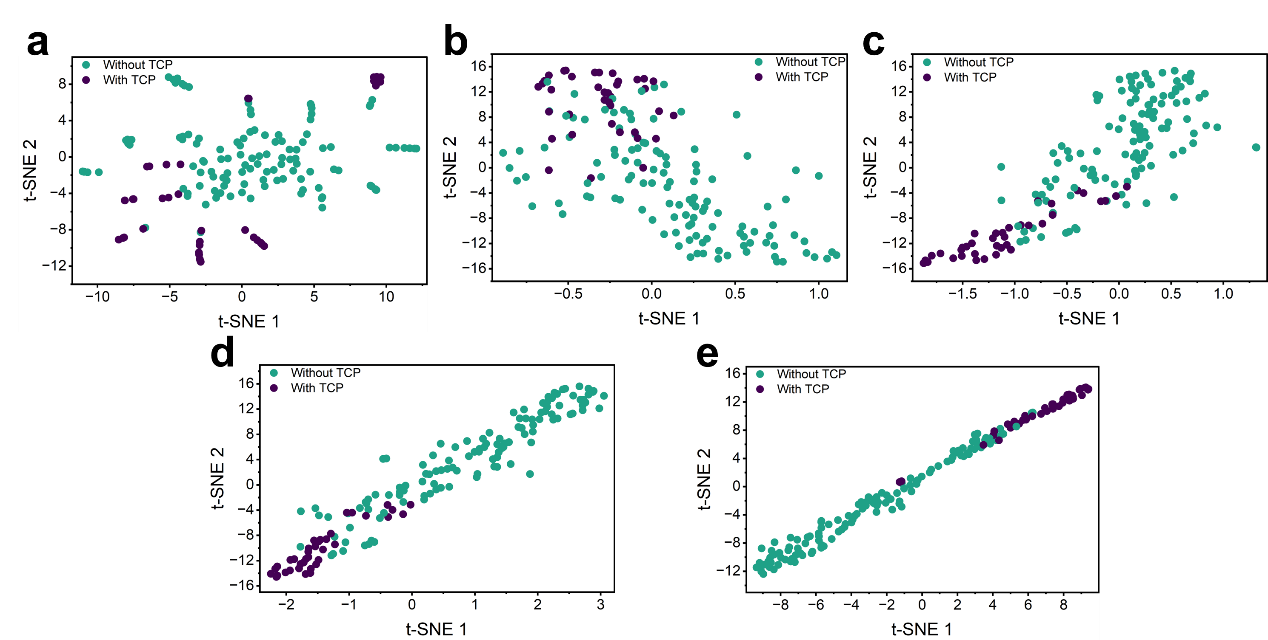


**Figure S9.** Visualization of hidden-layer outputs in the target domain model for TCP phase classification. a)-c) Frozen layers transferred from the source domain model. d)-e) Newly added and trained hidden layers.

We further analyzed the role of the newly added layer by evaluating the discriminative ability of its output features. The output vectors from the last frozen layer and the last newly added layer were separately extracted and used as inputs to logistic-regression classifiers. The two models constructed based on these features were evaluated on the extrapolation testing set. The classifier based on output vectors from the newly added hidden layer outperformed that based on the frozen layer in both accuracy and recall, as shown in Table S6. This indicates that the newly added hidden layers further improve the accuracy of the transferred representations while preserving the transferable knowledge learned from the source domain. Combined with the visualization of hidden-layer embeddings, these results suggest that the newly added layers help correct the residual source domain bias contained in the frozen representations.

**Table S6.** Confusion matrices and performance metrics of two transfer-learning strategies on the extrapolation testing set.

| Model | TP | FN | FP | TN | Accuracvy | Recall | Precision | F1 |
| --- | --- | --- | --- | --- | --- | --- | --- | --- |
| LR(frozen) | 67 | 14 | 9 | 15 | 78.10% | 82.72% | 88.16% | 85.35% |
| LR(added) | 70 | 11 | 7 | 17 | 82.86% | 86.42% | 90.91% | 88.61% |

For comparison, we further evaluated the proposed strategy against a transfer-learning approach based on progressive unfreezing. The source domain model was constructed in the same manner as described above, after which the original output layer was replaced with a new one. The model was then trained by gradually unfreezing layers in a layer-wise manner. The best predictive performance obtained on the extrapolation testing set is summarized in the Table S7.

**Table S7.** Confusion matrices and performance metrics of two transfer-learning strategies on the extrapolation testing set.

| Model | TP | FN | FP | TN | Accuracvy | Recall | Precision | F1 |
| --- | --- | --- | --- | --- | --- | --- | --- | --- |
| TL(this work) | 77 | 4 | 6 | 18 | 90.48% | 95.06% | 92.77% | 93.90% |
| TL(progressive-unfreezing strategy) | 72 | 9 | 12 | 12 | 80.00% | 88.89% | 85.71% | 87.27% |

The *R^2^* values for the *V*_γ′_, *S*_γ′_ and *N*_γ′_ models are shown in the Table S8.

**Table S8.** *R^2^* values for *V*_γ′_, *S*_γ′_ and *N*_γ′_ models.

| Model | Source Domain | Target Domain |
| --- | --- | --- |
| *V_γ′_* | Training Set: 1.00  Testing Set: 0.99 | Training Set: 0.98  Testing Set:0.94 |
| *S_γ′_* | Training Set: 0.99  Testing Set: 0.98 | Training Set: 0.97  Testing Set: 0.88 |
| *N_γ′_* | Training Set: 0.99  Testing Set: 0.95 | Training Set: 0.99  Testing Set: 0.95 |

The *R^2^* values for the *V*_γ′_, *S*_γ′_ and *N*_γ′_ models are shown in the Table S9.

**Table S9.** *R^2^* values for yield strength and the time of 0.2% creep strain models.

| Model | Training Set | Testing Set |
| --- | --- | --- |
| yield strength | 0.95 | 0.84 |
| the time of 0.2% creep strain | 0.99 | 0.89 |

**Discussion on the accuracy of the mechanical-property model**

In this work, the predictive uncertainty was estimated through a resampling-based approach, where multiple models were constructed and the variance of the predictions was used to quantify the uncertainty. In addition, both yield strength and the time to 0.2% creep strain were measured through repeated experiments, allowing the experimental variability (σ_E_) to be evaluated, as summarized in Table S10. By comparing the predicted mean values (μP) and their associated uncertainty (σ_P_) with the experimental results (μ_E_ ± σ_E_), it is observed that the experimental values fall within the predicted intervals (μ_P_ ± σ_P_). This indicates that the model uncertainty is reasonably calibrated, and the predictions are statistically consistent with the observed experimental variability. This indicates that the predicted uncertainty reasonably reflects the actual variation observed in the experiments.

**Table S10.** Comparison of predicted and experimental mechanical properties of USTB-PM750 with uncertainty quantification.

| Alloy | Predicted values μ_P_ | Uncertainty σ_P_ | Results from repeated experiments | Experimental values μ_E_ | Error σ_E_ | μ_E_∈[μ_P_-σ_P_, μ_P_ +σ_P_] |
| --- | --- | --- | --- | --- | --- | --- |
| Yield strength(MPa) | 1130 | 15 | 1131  1137  1146 | 1138 | 8 | Yes |
| The time of 0.2% creep strain (hours) | 134 | 38 | 126  141 | 134 | 9 | Yes |

To further evaluate the applicability of the model under various conditions, it was applied to predict the properties of Ni-based superalloys with different compositions developed by our group at different temperatures. The predictions were compared with the corresponding experimental measurements, as summarized in the following Table S11. The absolute percentage error was below 3.07% for yield strength and below 7.54% for the time to reach 0.2% creep strain. These results indicate that the model retains good predictive capability at other temperatures.

**Table S11.** Comparison between predicted and experimentally measured mechanical properties of Ni-based superalloys with different compositions under various conditions.

| Alloy | Test condition | Yield strength(MPa) | | Error (%) | Test condition | the time of 0.2% creep strain (hours) | | Error (%) |
| --- | --- | --- | --- | --- | --- | --- | --- | --- |
|  |  | Predicted values | Experimental values |  |  | Predicted values | Experimental values |  |
| 1# | 20 ℃ | 1259 | 1252 | 0.56 | 704℃ 690MPa | 649 | 675 | 3.85 |
| 2# | 20 ℃ | 1230 | 1269 | 3.07 | 704℃ 690MPa | 383 | 409 | 6.36 |
| 3# | 750 ℃ | 1099 | 1081 | 1.67 | 700℃ 730MPa | 271 | 252 | 7.54 |

**Comparison of PM Superalloy**

**Table S12** shows the superalloy yield strength and creep resistance of Ni-based PM superalloys. Compared with these alloys, the UTSB-PM750 alloy designed in this work exhibits superior overall high-temperature performance.

**Table S12.** Mechanical properties of Ni-based PM superalloys.

|  | Test condition | Yield strength (MPa) | Test condition | The time of 0.2% creep strain (h) |
| --- | --- | --- | --- | --- |
| Alloy 10 | 750 ℃ | 1065 | 750 ℃/480 MPa | 90 |
| ME3 | 750 ℃ | 1002 | 760 ℃/480 MPa | 239 (Larson-Miller extrapolated life) |
| LSHR | 750 ℃ | 1103 | 750 ℃/480 MPa | 49(Larson-Miller extrapolated life) |
| N18 | 750 ℃ | 1010 | 750 ℃/480 MPa | 25(Larson-Miller extrapolated life) |
| RR1000 | 750 ℃ | 996 | 750 ℃/460 MPa | 40 |
| U720Li | 750 ℃ | 940 | 750 ℃/350 MPa | 10 |
| FGH96 | 750 ℃ | 1040 | 750 ℃/480 MPa | 12 |
| FGH97 | 750 ℃ | 986 | 750 ℃/450 MPa | 157 |
| Alloy A | 750 ℃ | 1065 | 750 ℃/480 MPa | 87 |
| Alloy B | 750 ℃ | 1070 | 750 ℃/480 MPa | 58 |

**SHAP**

The effects of the key features identified by SHAP analysis on the performance models are shown in **Figure S10**.

**
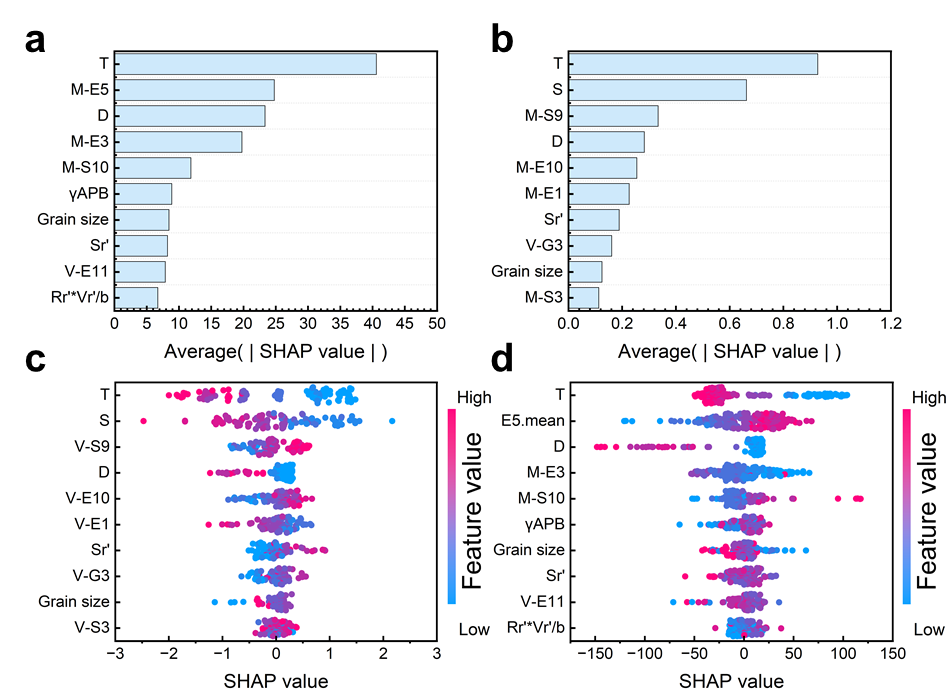
**

**Figure S10.** a) Feature importance ranking for the yield strength model and b) the time of 0.2% creep strain model. SHAP value summary plot for c) yield strength model and d) the time of 0.2% creep strain model.

**Microstructure of the UTSB-PM750 alloy after standard heat treatment**

**Figure S11** shows the microstructure of the UTSB-PM750 alloy after standard heat treatment, where the grain boundaries are serrated and annealing twins are observed within the grains. Coarse primary γ′ precipitates are distributed along the grain boundaries, while regularly distributed secondary γ′ precipitates are present within the grains.


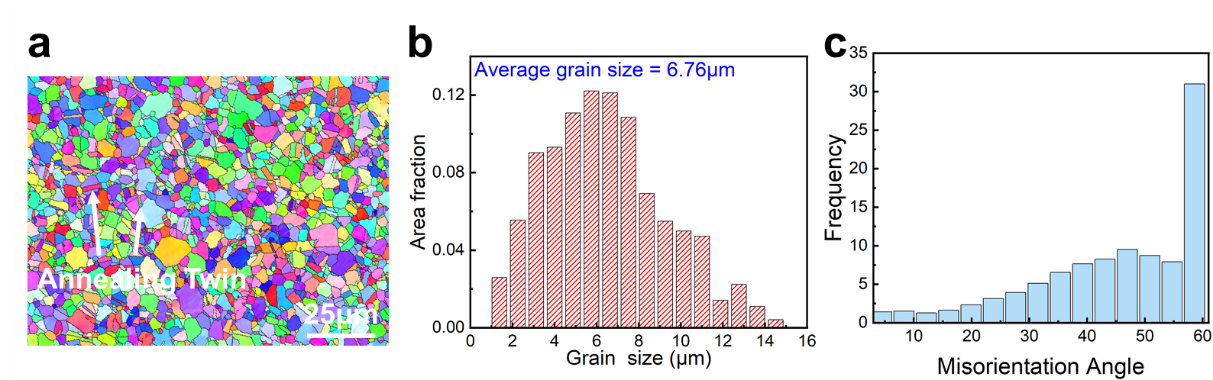


**Figure S11.** a) Microstructure of the USTB-PM750 alloy after standard heat treatment. b) Grain size. c) Grain boundary misorientation distribution.

**Elemental segregation at planar defects in the UTSB-PM750 alloy**

**Figure S12** presents the HRTEM images and atomic-resolution EDS elemental maps of superlattice extrinsic stacking fault (SESF) and microtwin (MT) formed in the UTSB-PM750 alloy during creep. At the SESF, elemental segregation is characterized by pronounced enrichment of Co together with moderate enrichment of W, Ta, and Nb, accompanied by clear depletion of Ni and Al. Similar segregation is also observed along MT boundaries, which is expected to retard MT thickening and thereby enhance the creep resistance of the alloy.

**
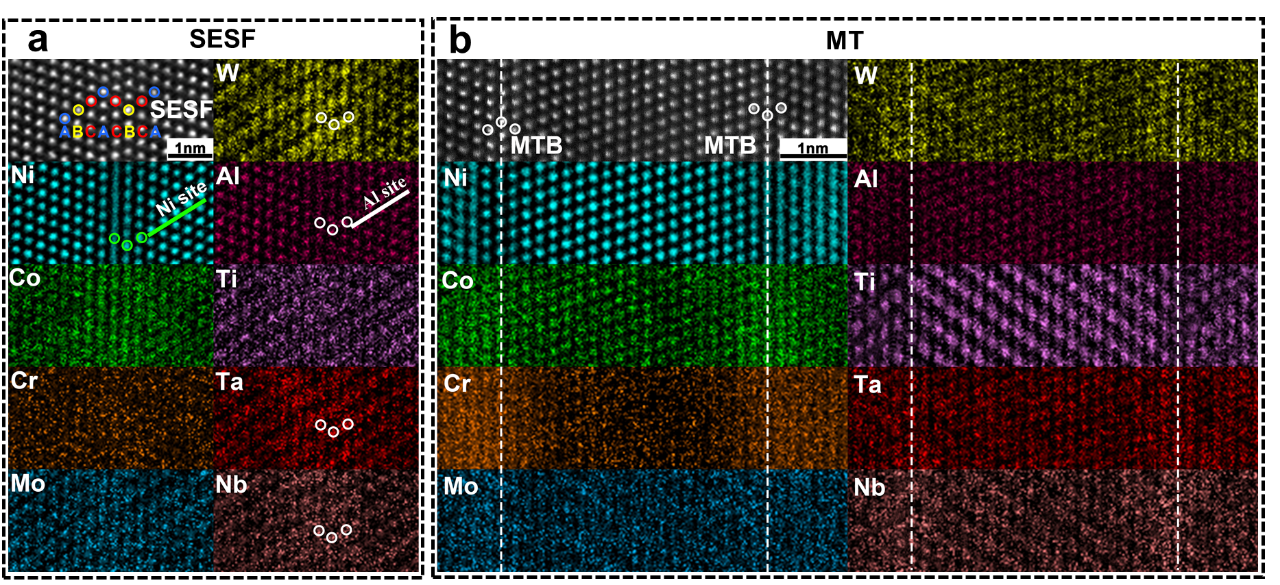
**

**Figure S12.** HRTEM images and atomic-resolution EDS elemental maps of a) SESF and b) MT.

**References**

[1] Y.-K. Kim, D. Kim, H.-K. Kim, C.-S. Oh, B.-J. Lee, Int. J. Plast. 2016, https://doi.org/10.1016/j.ijplas.2015.12.008.

[2] R. C. Reed, T. Tao, N. Warnken, Acta Mater. 2009, 57, 5898–5913. https://doi.org/10.1016/j.actamat.2009.08.018

[3] L. A. Gypen, A. Deruyttere, J. Mater. Sci. 1977, 12, 1028–1033. https://doi.org/10.1007/BF00540987

[4] R. C. Reed, Z. Zhu, A. Sato, D. J. Crudden, Mater. Sci. Eng. A 2016, 667, 261–278. <https://doi.org/10.1016/j.msea.2016.04.089>

[5] D. J. Crudden, A. Mottura, N. Warnken, B. Raeisinia, R. C. Reed, Acta Mater. 2014, 75, 356–370. <https://doi.org/10.1016/j.actamat.2014.04.075>
